# A systematic review of pragmatic language interventions for children with autism spectrum disorder

**DOI:** 10.1371/journal.pone.0172242

**Published:** 2017-04-20

**Authors:** Lauren Parsons, Reinie Cordier, Natalie Munro, Annette Joosten, Renée Speyer

**Affiliations:** 1 School of Occupational Therapy and Social Work, Curtin University, Perth, Western Australia, Australia; 2 Faculty of Health Sciences, The University of Sydney, Sydney, New South Wales, Australia; 3 College of Healthcare Sciences, James Cook University, Townsville, Queensland, Australia; TNO, NETHERLANDS

## Abstract

There is a need for evidence based interventions for children with autism spectrum disorder (ASD) to limit the life-long, psychosocial impact of pragmatic language impairments. This systematic review identified 22 studies reporting on 20 pragmatic language interventions for children with ASD aged 0–18 years. The characteristics of each study, components of the interventions, and the methodological quality of each study were reviewed. Meta-analysis was conducted to assess the effectiveness of 15 interventions. Results revealed some promising approaches, indicating that active inclusion of the child and parent in the intervention was a significant mediator of intervention effect. Participant age, therapy setting or modality were not significant mediators between the interventions and measures of pragmatic language. The long-term effects of these interventions and the generalisation of learning to new contexts is largely unknown. Implications for clinical practice and directions for future research are discussed.

## Introduction

A core characteristic of autism spectrum disorder (ASD) is a deficiency in social communication and interaction. A wide range of verbal language abilities are reported in individuals with ASD, but a striking feature about their language profile is a universal impairment in pragmatic language [1]. This review will focus on interventions that target the pragmatic aspect of language. Early definitions of pragmatic language refer to the use of language in context; encompassing the verbal, paralinguistic and non-verbal aspects of language [2]. Contemporary definitions have expanded beyond just communicative functions to include behaviour that includes social, emotional, and communicative aspects of language [3]. This expansion reflects an understanding that pragmatic language, social skills and emotional understanding are interconnected, and this definition of pragmatic language will be used for this review. While this definition encompasses pragmatics en masse, one of the challenges for a systematic review on pragmatic language interventions for children with ASD is identifying the skills of pragmatics that are actually targeted. The following sections therefore provide a brief summary of pragmatic language development, the skills identified as problematic in children with ASD and a framework for classifying interventions.

Pragmatic language behaviours emerge during the prelinguisitic phase of language development. Early language is typically characterised by a combination of gestures, vocalisations, and simple phonetic forms [4]. While linguistically simple, these acts are social in nature and are interpreted by adults as communicative in intent, leading to descriptions of children as “pragmatically precocious” [4]. Further, joint attention acts as a scaffold for the development of social communication [4]. Children with ASD display a lack of joint attention that begins in infancy, and therefore display developmental differences in related communicative acts, such as the use and comprehension of gestures, and attention to a social partner and a shared topic (joint engagement) [5]. Further, approximately 30% of individuals with ASD develop only minimal verbal communication [6], so interventions that target these early, preverbal stages of pragmatic language are developmentally important for children with ASD as they can enhance future language and social development [7].

During typical development, a range of communicative acts emerge and continue to develop as structural language develops, conversational topic maintenance emerges in interactions with adults, and the appropriateness of responses increases [1, 4]. The communicative, social and emotional aspects of pragmatic language have recently been described in 27 observable communicative behaviours, classified into five domains relevant for children aged 5–11 years [8]. The domains are: 1) Introduction and responsiveness (the ability to introduce communication and be responsive to the communication of others); 2) Non-verbal communication (the use and understanding of gestures, facial expressions, body postures and proximity between speakers); 3) Social-emotional attunement (interpreting the emotional reactions of others and demonstrating appropriate responses); 4) Executive function (attending to interactions and flexibility in planning communicative content); and 5) Negotiation (cooperating and negotiating appropriately with communicative partners). For children with ASD who develop verbal language, previously described pragmatic difficulties persist and further pragmatic language deficits evolve, including fewer and often unskilled attempts at initiating communication, narrower ranges of communication acts, and difficulties producing novel language [9].

Documentation about the typical progression of pragmatic language into adolescence is scarce. However, mastery of earlier emerging conversational skills such as cohesion, appropriate referencing, and providing adequate responses is reported, along with an equal distribution of conversational burden, and an ability to adapt speaking style to one’s conversational partner or context [10]. Despite the limited knowledge on what is typical in adolescence, some differences in pragmatic language competence in individuals with ASD have been reported, such as poor conversational topic management, the contribution of irrelevant information to conversations, unusual prosody, reduced reciprocity and responses to partner cues, and inappropriate eye-gaze [11].

In summary, deficits in pragmatic language affect individuals with ASD throughout childhood necessitating effective, evidence-based interventions that can minimise the isolating, and long-term impacts of pragmatic language difficulties. Two studies have reported increased feelings of loneliness and poorer friendship quality in children and adolescents with ASD when compared to typically developing peers as a result of reduced pragmatic language skills [12, 13]. Long-term outcomes have been studied in a sample of adults identified during childhood as having either a pragmatic language impairment (PLI) or ASD [14]. Participants with ASD were found to have substantial pragmatic difficulties that persisted into adulthood, and the quality of social relationships were poor for both adults with ASD and PLI. No participant in the ASD group reported any close friendships or romantic relationships.

A recent review of 26 spoken language intervention studies for children with ASD found a small effect on structural language competence [15], but to date there is no review of interventions that target pragmatic language in children and adolescents with ASD. The purpose of this study is to conduct a systematic review and meta-analysis of pragmatic language interventions for children with ASD. The review will describe the studies reporting on pragmatic language interventions for children with ASD and the characteristics of the included interventions, and evaluate the methodological quality of the included studies. A meta-analysis will be conducted to answer the following research questions: 1) do different settings (i.e., home, clinic, or school), person(s) of focus (i.e., child, parent, or both), or intervention modalities (i.e., individual, group, or both) produce different intervention effects?; 2) are pragmatic language interventions more effective than no treatment or usual treatment practices?; and 3) do participant age, type of outcome measure, or the aforementioned intervention characteristics mediate intervention effect?

## Methods

The PRISMA statement guided the methodology and reporting of this systematic review and the review was registered with the PROSPERO register of systematic reviews (registration number CRD42015029161). A completed PRISMA checklist is provided in S1 Table.

### Information sources

A comprehensive literature search was initially conducted using subject headings and free-text strings across five electronic databases on April 8, 2015. An updated free-text search of the same databases was conducted on May 14, 2016 to capture any new papers published since the original search. The databases searched were: CINAHL, Embase, Eric, PsychINFO and PubMed. A Google Scholar search was also conducted on November 26, 2015, and a search within autism focused journals was conducted on November 30, 2015 in order to identify any additional articles. The speechBITE website (www.speechbite.com), a database of intervention studies in the field of speech pathology created and maintained by an advisory committee based in the Discipline of Speech Pathology at The University of Sydney, was searched for interventions pertaining to pragmatics/social communication for children in the ASD population. Evidence-based Practice Briefs published on SpeechandLanguage.com (www.speechandlanguage.com/ebp-briefs) were searched. SpeechandLanguage.com is a professional development focused site for speech pathologists maintained by Pearson. Finally, reference lists of included articles were searched to identify additional studies.

### Search strategy

In searching electronic databases two search categories were combined: 1) fields in language studies (pragmatics, social language, social communication, paralinguistics, nonverbal communication, prosody, social behaviour, social skills, communication, communication disorders, child language, verbal behaviour, language, language tests, language therapy, language development disorders, speech therapy) and 2) disorder (autism, autism spectrum disorder, autistic disorder, pervasive developmental-disorder not otherwise specified, Asperger syndrome, Rett syndrome, child disintegrative disorder). As no database contained a subject heading related to pragmatic language, more general terms in the field of language and social skills were included in an attempt to capture all literature on the subject; thus casting a wide net. Limitations were applied for participant age (0–18 years), and English language. Free text searches were also conducted in all databases for papers published between April 8, 2014 and May 14, 2016. The full search strategy, including subject headings, free-text and limitations for each database is provided in S2 Table.

### Eligibility criteria

As pragmatic language difficulties present at a very young age in children with ASD and persist into adulthood, it is necessary for therapists to provide pragmatic language interventions to children throughout their development. This review will therefore assess the range of interventions available to address pragmatic language difficulties through childhood and adolescence. In order to classify pragmatic language skills for the purpose of this review, the five domains of Introduction and Responsiveness, Non-verbal Communication, Social-emotional Attunement, Executive Function and Negotiation are used as a framework [8]. While the pragmatic language behaviours that these domains encompass are indented for children aged 5–11 years, the pragmatic behaviours of early intentional communication observed in children younger than five years are nonetheless subsumed within the domains (e.g., uses and responds to a variety of gestures, initiates verbal communication, responds to the communication or others). This was deemed the most appropriate contemporary framework to utilise in the absence of a pragmatic language classification system that adopts a developmental approach.

To be included in the review, articles were required to meet the following criteria: 1) participants were children (aged 0–18 years) with a primary diagnosis of ASD (including Asperger syndrome, or PDD-NOS for children diagnosed prior to the Diagnostic and Statistical Manual of Mental Disorders (DSM)—Fifth Edition), with or without an intellectual disability; 2) treatment focused on preverbal pragmatic language behaviours or at least one of the behaviours broadly encompassed by the pragmatic language domains of pragmatic language domains of Introduction and Responsiveness, Non-verbal Communication, Social-emotional Attunement, Executive Function and Negotiation; 3) studies included a control group with random assignment to groups; 4) treatment outcomes measured at least one of the skills encompassed by the definition of pragmatic language adopted for this review. Only papers published in English in peer reviewed journals were considered for this review. Pharmacological interventions were excluded. Outcome measurements of autism symptom severity were not considered assessments of pragmatic language for the purpose of this review. These criteria were used in order to identify all randomised controlled trials of pragmatic language interventions for children with ASD.

### Systematic review

#### Methodological quality

The Standard Quality Assessment criteria for evaluating primary research papers from a variety of fields (Kmet checklist) was used to assess the methodological quality of the included studies [16]. The 14-item checklist utilises a 3-point, ordinal scale (0 = no, 1 = partial, 2 = yes), giving a systematic and quantifiable means for assessing the quality of studies of a variety of research designs [16]. Checklist items assess the sampling strategy, participant characteristics described, sample size calculations, sample size collection, description and justification of analytic methods, result reporting, controls for confounding variables, and whether conclusions drawn reflect results reported. An overall quality percentage score can be calculated by dividing the total score rated by the maximum possible score, and studies were then classified based on that score. The following convention was used for the classification of methodological quality [17, 18]: a score of >80% was considered strong quality, a score of 70–79% was considered good quality, 50–69% was considered fair quality and <50% was considered to have poor methodological quality.

#### Data collection process

Comprehensive forms were developed in order to extract relevant data from the included studies. Data on study characteristics were extracted for the following categories: participant diagnosis, control group, age range (mean and standard deviation), study eligibility criteria, treatment condition, outcome measures and treatment outcomes. Extraction of data pertaining to intervention components was guided by the TIDieR Checklist, a 12-item checklist that guides the reporting of intervention studies so that procedures can be replicated by other researchers and clinicians in the field [19]. Data were extracted for skill(s) targeted, materials and procedures, interventionists, duration and setting/mode of delivery, tailoring/modifications, methods of blinding and randomisation. Data relating to methodological quality were extracted in accordance with the Kmet checklist.

#### Data items, risk of bias and synthesis of results

All abstracts were reviewed by one researcher for inclusion, and a second researcher reviewed a randomly selected 40% of the abstracts to ensure accuracy in study selection for the review. The same assessors also rated the extracted data pertaining to methodological quality of all included studies using the Kmet checklist. Interrater reliability between the two independent assessors was established for both the abstract selection and Kmet ratings of each included study. The likelihood of bias was reduced in the extraction of data and in ratings of study quality for this review, as none of the reviewers have any affiliations with any of the authors of the included studies. Data was synthesised and summarised into a number of categories including study design, participant characteristics, inclusion criteria, treatment components and outcomes, and methodological quality. Treatment effectiveness was assessed using significance values and effect sizes of the main pragmatic language outcome measure.

### Meta-analysis

Subsampling was chosen as the predominant analytic technique for this review, as the small number of included studies limited the viability of meta-regression using multiple covariates. Data was extracted from the included studies to measure the overall effect of pragmatic language interventions for children with ASD, and treatment effect as a function of the following intervention characteristics: 1) setting (i.e., home, school or clinic); 2) focus of the intervention (i.e., child, parent and child, parent only), and; 3) the mode of delivery (i.e., individual or group). An analysis of the interventions based on the pragmatic language skills targeted was considered; however, grouping interventions in this way would cause a comparison of a large number of small groups, thus limiting the conclusions that could been drawn from the results.

Meta-regression was conducted to determine whether participant age, type of outcome measure, or any of the three aforementioned intervention components mediated intervention effect. The study sample size (17) allowed for multivariate analysis involving up to two covariates without compromising power [20], so one multivariate model addressed the interaction between participant age and mode of intervention delivery. This model was selected as participant age potentially confounded the results of the subgroup analysis pertaining to mode. Lastly, between-groups analyses assessed the difference in post-intervention social communication competence of those who received a pragmatic language intervention and their comparison controls who were groups by condition type (i.e., no treatment, treatment as usual, or an alternative treatment).

To compare effect sizes, pre- and post- means, standard deviations, and sample sizes were extracted. If the data required for meta-analysis calculations was not reported, attempts were made to contact authors in order to request the desired data. In cases where more than one paper reported on the same study sample, the paper reporting an outcome measure that evaluated the greatest number of pragmatic language skills covered by the definition adopted for this review was chosen for the analysis. Studies reporting on follow-up data only were also excluded. When multiple outcome measures of social communication were reported for one intervention, the measure that evaluated the greatest number of pragmatic language skills was extracted for analysis. If a single outcome measure could not be chosen, then means for multiple measures of pragmatic language were averaged and pooled standard deviations were calculated for the meta-analysis.

Extracted means, standard deviations, and sample sizes for pre- and post- measures were entered into Comprehensive Meta-Analysis, Version 3.3.070. A random effects model was used to generate effect sizes as the included studies are not likely to have the same true effect due to the variability in the sampling, intervention characteristics, skills targeted, participant characteristics and outcome measures utilised.

Heterogeneity was estimated via two methods. The *Q* statistic determines the spread of all effect sizes around the mean effect size. As *Q* can be poor at detecting heterogeneity in analyses with low power, *I*^*2*^ was also examined [21]. The *I*^*2*^ statistic estimates the ratio of true variance to total variance. For all sub-group analyses the Hedges *g* formula for standardised mean difference (SMD) with a confidence interval of 95% was used to report effect sizes. Using Cohen’s *d* convention for interpretation, an effect size of ≤0.2 reflects negligible difference, between ≥ 0.2 and ≤ 0.49 was considered as small; between ≥ 0.5 and ≤ 0.79 was considered as moderate; and ≥ 0.8 was considered as large [22].

Given that studies that report large and significant treatment effects are more likely to be selected for publication, it is possible that some low-effect or non-significant interventions are missing from the meta-analysis. The presence of publication bias was assessed using classic fail-safe N. The test calculates the number of additional studies that, if added to the analysis, would nullify the measured effect (N). If N is large it can be considered unlikely that there would be so many unpublished low-effect studies and it can be assumed that the meta-analysis is not compromised by publication bias.

## Results

### Study selection

A total of 2,909 papers were identified through the initial subject heading and free text searches across the following databases: CINAHL, Embase, Eric, PsychINFO and PubMed. A further 29 records were identified via Google Scholar, autism specific journals, speechBITE, and SpeechandLanguage.com. These 2,938 studies were screened for duplicate titles and abstracts and 840 duplicated records were removed. The updated database search added a further 793 unique abstracts for screening. Two reviewers rated abstracts for inclusion. The first author assessed all 2,891 eligible abstracts against the inclusion criteria, with a randomly selected 40% of the studies assessed by a second rater for inter-rater reliability. The agreement between raters measured by Weighted Kappa was 0.84 (95%CI: 0.66–1.00). There were only three abstracts in the random selection on which the raters did not agree, so all three records were included for further full text screening.

**Fig 1:**
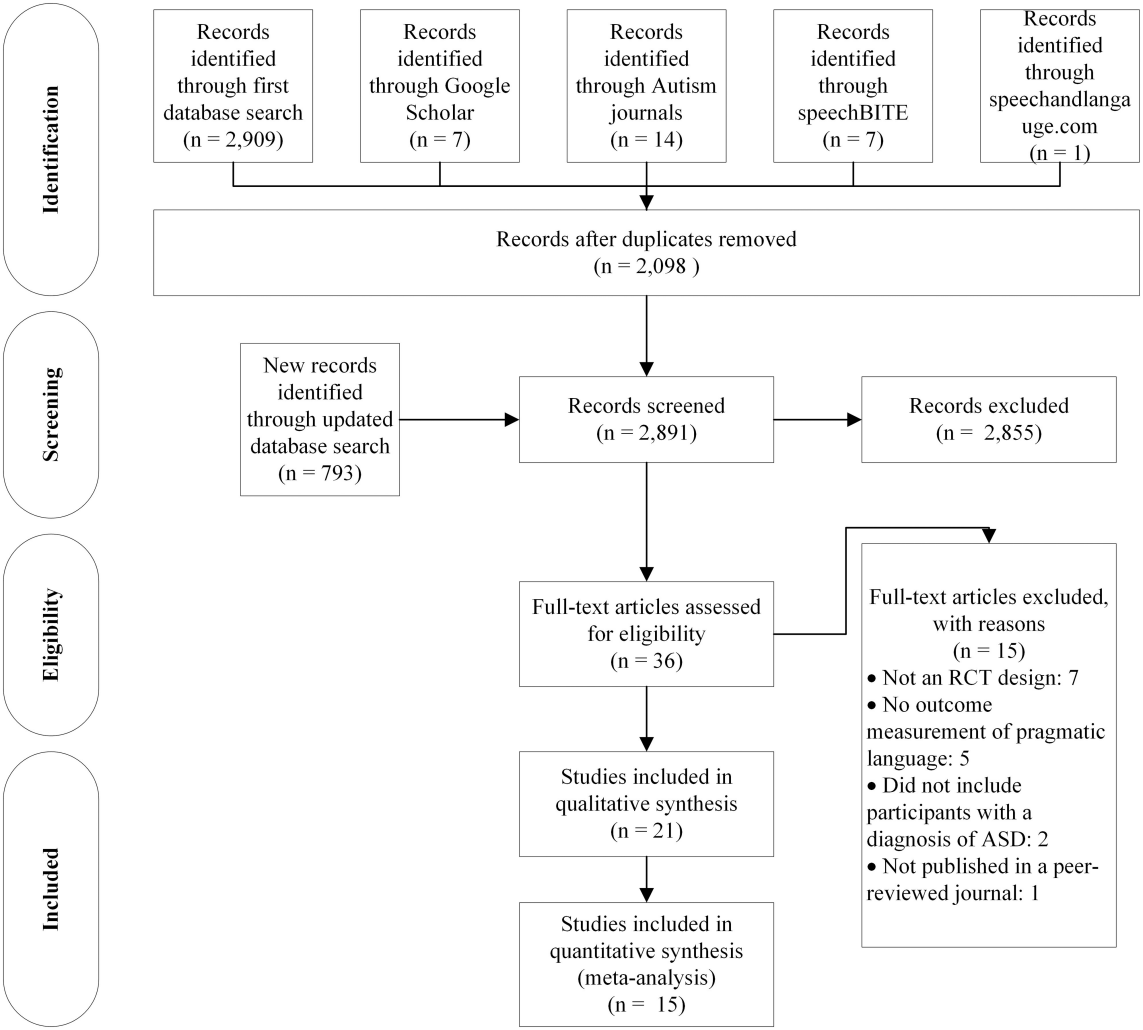
Study flow diagram.

After assessing abstracts on the criteria for inclusion a total of 36 studies were identified. Full text records were accessed via Curtin University and the University of Sydney libraries to further determine whether the studies met the criteria for inclusion in this review. Of these 36 studies, seven were not randomised controlled trials, five did not have an outcome measurement that assessed pragmatic language, two did not include participants with ASD, and one was not published in a peer reviewed journal (Fig 1). References for the 15 studies excluded and reasons for exclusion are presented in Table 1. A total of 21 papers, reporting on 18 different intervention studies were selected for inclusion based on the inclusion criteria (See Fig 1). All of the included studies used a randomised controlled design, included participants aged 0–18 years with a diagnosis of ASD, and performed an intervention that aimed to improve any of the pragmatic language skills incorporated by the definition of pragmatic language adopted for this review.

**Table 1:** Excluded studies with reasons for exclusion.

Study	Reason for exclusion
Gattino, dos Santos Riesgo [23]	No outcome measurement that assessed pragmatic language
Ichikawa, Takahashi [24]	No outcome measurement that assessed pragmatic language
Kasari, Rotheram-Fuller [25]	No outcome measurement that assessed pragmatic language
Lerner and Mikami [26]	No outcome measurement that assessed pragmatic language
Wong and Kwan [27]	No outcome measurement that assessed pragmatic language
Houghton, Schuchard [28]	Not a randomised controlled trial
McFadden, Kamps [29]	Not a randomised controlled trial
McMahon, Vismara [30]	Not a randomised controlled trial
Oosterling, Visser [31]	Not a randomised controlled trial
Radley, Ford [32]	Not a randomised controlled trial
Shire, Goods [33]	Not a randomised controlled trial
Wetherby, Guthrie [34]	Not a randomised controlled trial
Adams, Lockton [35]	Participants did not have a core diagnosis of ASD
Kamps, Thiemann-Bourque [36]	Participants did not have a core diagnosis of ASD
Donaldson [37]	Not published in a peer reviewed journal

### Description of studies

Tables 2–5 include a detailed description of the included studies. Data points were collected and synthesised as follows: Intervention studies for improving pragmatic language in children with ASD (Table 2), intervention components (Table 3), pragmatic language skills targeted (Table 4), and the methodological quality of included studies (Table 5).

**Table 2:** Characteristics of included studies.

Treatment/Target Skills	Reference, Location	Participant groups (N)	Age Years (Mean ± SD)	Inclusion/Exclusion Criteria	Pragmatic Language Outcome Measure	Treatment Outcome
**The Junior Detective Program (TJDP):** Emotion recognition through gesture, posture, prosody; Initiating and maintaining a conversation	Beaumont and Sofronoff [41] Australia	Treatment: 26	9.64 ± 1.21	*Inclusion*: ASD diagnosis, Short form WISC-III score ≥85, Aged 7 ½-11 years	Assessment of Perception of Emotion from Facial Expression	Significant improvement in both groups. No significant difference between groups.
		Control: 23	9.81 ± 1.26		Assessment of Perception of Emotion from Posture Cues	Significant improvement in both groups. No significant difference between groups.
**Milton & Ethel Harris Research Initiative (MEHRI) treatment:** Engage in conversations or proto-conversations; Use ideas and language functionally	Casenhiser, Shanker [42] Canada	Treatment: 25	3.54 ± 0.73	*Inclusion*: ASD diagnosis; Chronological age 24–59 months	mCBRS: Initiation of Joint Attention subscale	Improvements made by intervention group were significantly higher than controls.
		Control: 26	3.87 ± 0.69			
	Casenhiser, Binns [43] Canada	See Casenhiser, Shanker [42]	See Casenhiser, Shanker [42]	See Casenhiser, Shanker [42]	25-min child-parent play session coded for response types (response to comments, obligatory responses, and contingent responses)	Treatment group made significantly greater increase in use of obligatory and contingent responses. No significant difference between groups in production or responses to comments.
					25-min child-parent play session coded for communicative acts (commenting, labelling, responding, directing, sharing, obtaining information, rejecting or protesting, social conventions and routines, spontaneous social expressions)	Post-hoc analysis showed significant time x group interactions for: commenting, labelling, sharing, obtaining information, rejecting or protesting.
**Building Blocks program—home based (HB):** Functional communication	Roberts, Williams [44] Australia	HB: 27	3.45 (range 2.2–4.95)	*Inclusion*: ASD diagnosis; Pre-school aged at start of program; Able to access centre for treatment	The Pragmatics Profile	Statistically significant changes pre to post intervention in all groups. No statistically significant differences in changes made between-groups.
**Building Blocks program—centre based (CB)**: Functional communication		CB: 29	3.59 (range 2.2–5)			
		Control: 28	3.64 (range 2.3–5.0)			
**Social Emotional NeuroScience Endocrinology (SENSE) Theater:** Engage in directed communication; Use gestures and nonverbal communication in directed ways; Empathic responding	Corbett, Key [45] USA	Treatment: 17	11.27 ± 2.51	*Inclusion*: *ASD* diagnosis; WASI score ≥70	SRS—Social Communication Scale	Significant difference measured between groups post treatment and at two month follow up.
		Control: 13	10.74 ± 1.89			
**Social Skills Group Intervention—High Functioning Autism (S.S.GRIN-HFA):** Non-verbal communication; Listening skills to effectively facilitate conversation	DeRosier, Swick [46] USA	Treatment: 27	10.2 ± 1.3	*Inclusion*: ASD diagnosis; Aged 8–12 years; WISC-IV verbal IQ score ≥85. *Exclusion*: CBCL T-score >70	SRS—Social Communication Scale	Significant treatment effect.
		Control: 28	9.9 ± 1.1			
**FindMe App:** Attending to people; Following social cues	Fletcher-Watson, Petrou [47] Scotland	Treatment: 27	4.12 ± 0.91	*Inclusion*: Diagnosis of ASD or on the waitlist for diagnosis; Aged <6 years at intake; Met Autism “cut-off” on social-communication algorithm of ADOS; Absence of neurological disorder; English speaking parents	Brief observation of social communication change (BOSCC)–Social Communication Scale	Not measured immediately following intervention. No significant difference between changes made by both groups at 6 month follow up.
		Control: 27	4.16 ± 1.1		CSBS-DP—Social Composite	No statistically significant difference found in change scores between groups baseline to post, or baseline to 6 month follow-up.
**Therapeutic Horse Riding:** Joint attention; Nonverbal communication	Gabriels, Pan [48] USA	Treatment: 58	10.5 ± 3.2	*Inclusion*: Aged 6–16 years; ASD diagnosis; SCQ score ≥15; Met clinical cut-off for ASD on ADOS; Irritability and Stereotypy subscales of the ABC-C combined score ≥11; Leiter-R nonverbal IQ standard score ≥40. *Exclusion*: Previously identified genetic disorder of a phenotype similar to ASD; History of medical or behavioural issues; History of animal abuse or phobia of horses; > 2 hours of equine assisted therapy within the past 6 months; Weight exceeding the riding centre’s policies	SRS—Social Communication Scale	Significantly greater improvement made by treatment group
		Control: 58	10.0 ± 2.7			
**FaceSay:** Responding to joint attention	Hopkins, Gower [49] USA	Treatment LFA: 11	10.31 ± 3.31	Not specified	Two five-minute observations of the children at school recess. Interactions coded for positive, negative and low-level initiations of social behaviour as per Hauck, Fein [50]	Significant difference in total score, and negative interactions score between LFA groups and HFA groups following intervention.
		Treatment: HFA: 13	10.57 ± 3.2			No significant difference in Positive Interactions or Low-level Interactions scores between LFA groups and HFA groups following intervention.
		Control: LFA: 14	10.05 ± 2.30		Emotion recognition of photographs and schematic drawings	Significant difference between change scores in LFA groups for total and photos only scores.
		Control: HFA: 11	9.85 ± 2.87			Significant difference between change scores in HFA groups for all scores (total, photos only and drawings only).
**Modified JASPER Intervention—Teacher delivered:** Initiation of Joint attention (point, show give)	Kaale, Smith [51] Norway	Treatment: 32	4.06 ± 0.69	*Inclusion*: ASD diagnosis; Chronological age 24–60 months; Attendance in preschool. *Exclusion*: CNS disorders; Non-Norwegian speaking parents	Frequency of JA initiation during ESCS	No significant difference between groups difference in changes measured.
		Control: 27	4.19 ± 0.69		Frequency of JA initiation during teacher-child play	Significant between groups difference in changes measured.
					Frequency of JA initiation during mother-child play	No significant difference between groups difference in changes measured.
					Duration of JE during teacher-child play	No significant difference between groups difference in changes measured.
					Duration of JE during mother-child play	Significant between groups difference in changes measured.
	Kaale, Fagerland [52] Norway	See Kaale, Smith [51]	See Kaale, Smith [51]	See Kaale, Smith [51]	Frequency of JA initiation during ESCS	No significant between groups difference in changes between baseline and 12 month follow-up.
					Frequency of JA initiation during teacher-child play	Significant between groups difference in changes from baseline to 12 month follow up.
					Frequency of JA initiation during mother-child play	No significant between groups difference in changes between baseline and 12 month follow-up.
					Duration of JE during teacher-child play	No significant between groups difference in changes between baseline and 12 month follow-up.
					Duration of JE during mother-child play	Significant between groups difference in changes from baseline to 12 month follow up.
**JASPER Intervention:** Initiations of joint attention (point, show, give); Response to joint attention	Kasari, Freeman [53] USA	Treatment: 20	3.6 ± 0.59	Not specified	Initiation of Joint Attention (showing, coordinated joint looks, pointing, giving) during ESCS	Treatment and Symbolic play groups showed greater improvement in showing than control group. No significant difference in showing between treatment and symbolic play groups. All groups showed significant improvement in coordinated joint looks. No significant differences noted in pointing or giving.
		Symbolic play: 21	3.5 ± 0.58			
		Control: 17	3.5 ± 0.41			
	
					15-minute caregiver-child interaction coded for joint attention skills: child’s frequency of joint attention skills (e.g., coordinated looks, pointing, and showing); time spent jointly engaged and interactive around objects; who initiated joint engagement (parent or child)	Treatment and Symbolic play groups showed significantly greater gains than the control group in coordinated joint looks. No significant difference in coordinated joint looks between treatment and Symbolic play groups. All groups showed significant improvement in pointing. Significant interaction effects found for pointing and showing (p<0.05). Treatment group showed significantly greater gains than the control group in child initiated joint attention. No differences were found in mother initiated joint engagement.
	Lawton and Kasari [54] USA	See Kasari, Freeman [53]	See Kasari, Freeman [53]	See Kasari, Freeman [53]	Shared positive affect during joint attention	No significant difference between treatment and Symbolic play groups at post, 6-month follow-up or 12-month follow-up.
					Shared positive affect with utterances during joint attention	No significant difference between treatment and Symbolic play groups at post, 6-month follow-up or 12-month follow-up.
**Modified JASPER Intervention—Parent-child dyad focused:** Initiating joint engagement; Initiating communication	Kasari, Gulsrud [55] USA	Treatment: 19 Control: 19	2.53 ± 0.08 2.61 ± 0.07	*Inclusion*: Aged <36 months; Meets ASD diagnostic criteria; No additional syndromes	15 minute caregiver-child interaction coded for joint attention (initiations and responses)	Significantly greater gains in responsiveness to joint attention for the treatment group (p<0.05). No significant differences measured in initiations of joint attention.
					15 minute caregiver-child interaction coded for engagement states (unengaged/other engagement, object engagement, joint engagement)	Treatment group made significant reductions in time spent in object engagement compared to the controls (p<0.01). Treatment group made significant increases in time spent jointly engaged compared to controls (p<0.05). No significant difference between groups in unengaged/other engagement states.
**JASPER—Caregiver Education Module:** Joint engagement with caregiver	Kasari, Lawton [56] USA	Treatment: 48 at exit, 44 at follow-up	3.5 ± .83	*Inclusion*: ASD diagnosis; “Low-resourced family”; Aged between 2 and 5 years; Mullen mental age >12 months.	ESCS—Initiation of joint attention skills	Both groups showed significant improvements immediately following intervention period (p<0.001). Gains for CMM group significantly greater than CEM group following intervention period (p = 0.05). Effect of treatment maintained for both groups at 12 week follow-up (p = 0.05).
		Control: 59 at exit, 51 at follow-up	3.57 ± .85			
					10 minute caregiver-child interaction coded for time spent jointly engaged	Both groups showed significant improvements immediately following intervention period (p<0.001). Gains for CMM group significantly greater than CEM group following intervention period (p<0.003). Treatment effect maintained for CMM group at 12-week follow-up (p = 0.02), but not the CEM group.
**Improvisational music therapy:** Joint attention behaviours (eye-contact, turn taking)	Kim, Wigram [57] Korea	Group one: 5 Group two: 5	All participants: 4.27 ± 1.0	*Inclusion*: ASD diagnosis	ESCS	Significant time x group interaction, with greater gains made post music therapy compared to post play sessions.
**SummerMAX + Mind Reading:** Social-communication; Face-emotion recognition	Lopata, Thomeer [39] USA	Treatment: 18 Control: 18	8.83 ± 1.47 8.83 ± 1.50	*Inclusion*: ASD diagnosis; WISC-IV short form IQ >70; WISC-IV VCI or PRI ≥80; Expressive or receptive language on the CASL ≥ 80	Cambridge Mindreading Face-Voice Battery for Children (CAM-C)	Significant time x treatment condition effect favouring SummerMAX + Mind Reading group for Faces score only.
					Emotion Recognition Display Survey (ERDS)	Statistically significant changes measured in all groups. No statistically significant differences in changes made between-groups.
					Social Emotional Evaluation	Statistically significant changes in Receptive scores measured in all groups. No statistically significant differences in changes made between-groups.
**Skillstreaming:** Face-emotion recognition	Lopata, Thomeer [38] USA	Treatment: 18 Control: 18	9.39 ± 1.72 9.56 ± 1.54	*Inclusion*: HFASD diagnosis; WISC-IV short form IQ >70; WISC-IV VCI or PRI ≥80; Expressive or receptive language on the CASL ≥ 80	DANVA2	ANCOVA results became non-significant after application of Bonferroni correction.
**Emotion Recognition Training:** Emotion recognition through facial expression	Ryan and Charragain [58] Ireland	Treatment: 20 Control: 10	9.25 ± 1.83 10.58 ± 2.08	*Inclusion*: Not specified. *Exclusion*: ERT score <80%; Difficulty with comprehension of emotion labels	ERT	Improvements made by the treatment group were significantly larger than those of the controls. Gains were maintained at 3 month follow-up for 25 participants measured.
**Seaver-NETT:** Nonverbal communication; Emotion recognition	Soorya, Siper [59] USA	Treatment: 35 Control: 34	10.05 ± 1.27 9.87 ± 1.32	*Inclusion*: ASD diagnosis; Aged 8–11 years; Verbal IQ score >70. *Exclusion*: Commencement of psychiatric medication 30 days prior to screening; Known gross structural abnormalities of the brain; Active seizure disorder; Aggression towards others	Social behaviour composite comprised of the following: SRS, CCC-2, and Griffith Empathy Measure	Statistically significant improvements following intervention compared to the control group. No significant in improvements measured between groups at 12-week follow-up.
**Mind Reading** [60]: Facial expression decoding; Prosody decoding	Thomeer, Smith [40] USA	Treatment: 22 Control: 21	8.57 ± 1.16 8.86 ± 1.39	*Inclusion*: ASD diagnosis; WISC-IV short form IQ >70; WISC-IV VCI or PRI ≥80; CASL short form expressive or receptive score ≥80	Cambridge Mindreading Face-Voice Battery for Children (CAM-C)	Intervention group had significantly higher Face and Voice scores than controls at post-test and 5-week follow-up.
					Emotion Recognition Display Survey (ERDS)	Intervention group had significantly higher Expressive scores than controls at post-test, and significantly higher Expressive and Receptive scores at 5-week follow-up.
					SRS	Intervention group had significantly lower scores (i.e. fewer symptoms) than controls at 5-week follow-up but not post-test.

*Notes*: RCT = Randomised Controlled Trial, ASD = Autism Spectrum Disorder, WISC-III = Wechsler Intelligence Scale for Children (Third Edition), mCBRS = Modified Child Behavior Rating Scale, PLS = Preschool Language Scale IV, CASL = Comprehensive Assessment of Spoken Language, MLUm = Mean Length of Utterance in morphemes, WASI =, SRS =, ABAS =, NEPSY = A Developmental NEuroPSYchological Assessment, MFI = Memory for Faces Immediate, MFD = Memory for Faces Delayed, TOM = Theory of Mind, WISC-IV = Wechsler Intelligence Scale for Children (Fourth Edition), SRS = Social Responsiveness Scale, ESCS = Early Social Communication Scales, ADOS = Autism Diagnostic Observation Schedule, CSBS-DP = Communication and Symbolic Behavior Scales—Developmental Profile, SCQ = Social Communication Questionnaire, ABC-C = Aberrant Behavior Checklist—Community, DANVA-2 = Diagnostic Analysis of Nonverbal Accuracy (Second Edition), ERT = Emotion Recognition Test, CCC-2 = Children’s Communication Checklist (Second Edition), SCERTS = Social Communication, Emotional Regulation, Transactional Supports, MSEL = Mullen Scale of Early Learning

**Table 3:** Pragmatic language intervention characteristics.

Intervention/Pragmatic Language Skills Targeted	Procedure	Interventionists	Duration and Setting/Mode of delivery	Tailoring/Modifications
**TJDP** [41]	Parents trained in use of TJDP (computer game facilitating practice in decoding emotions from non-verbal cues, and selecting appropriate reactions). Parent facilitates child’s use of TJDP. Children participate in group activities to generalise TJDP content and learn additional social and problem-solving skills. Parents attend concurrent training in skills that children are learning. Detection of emotions via non-verbal cues, practice of relaxation techniques, ‘play dates’ with peers and completion of ‘Secret Agent Journal’ completed at home. Token economy used in session to reward appropriate behaviour and completion of home practice.	*Therapist*: Postgraduate clinical psychology and counselling students, and the chief investigator; *Parent*	*14 hours + home practice*: 1 x 2-hour clinic session/week for 7 weeks. *Clinic*: computer program, group therapy (3 children and 2 therapists), parent training; *Home*: home practice	None described.
Emotion recognition through gesture, posture, prosody				
Initiating and maintaining a conversation				
**MEHRI Treatment** [42, 43]	No description of intervention materials or techniques provided. Therapists coached families on how best to facilitate interaction and communication with their child. First hour spent with one therapist, then 15–20 minute break for child while therapist consulted with parent regarding the therapy. Final hour spent with a second therapist. Caregiver spends 3 hours per day interacting with child away from clinic, and met with therapist to discuss progress and review videotapes child-caregiver play sessions every 8 weeks.	*Therapists*: speech pathologists and occupational therapists	*104 hours in clinic; 1095 hours at home*: 1 x 2-hour clinic session/week for 12 months; 3 home practice hours/day. *Clinic*: one-on-one therapy; *Home*: parent interaction with child at home	Each child assessed by therapist and strategies appropriate for the individual child and family identified to address strengths and challenges. Intervention identifies 5 developmental capacities and therapists attempt to ensure children are functioning adequately at lower capacities before targeting later capacities.
Engage in conversations or proto-conversations				
Use ideas and language functionally				
**Building Blocks program—centre based** [44]	Therapists facilitated manualised intervention with children. Session procedures or focus skills not described. Therapeutic techniques included direct intervention and less directed routines. Parent meetings operated concurrently, allowing parents to meet with professionals and other parents, and to form a support network. Topics included positive behaviour support, communication, self-help issues, school options, specialist services, and sensory issues.	*Therapists*: teachers, speech pathologists, occupational therapists, psychologists	*80 hours for child; 120 hours for parent*: 1 x 2-hour clinic session/week for 40 weeks; 1 x 3-hour parent sessions/week for 40 weeks. *Clinic*: Group therapy (4–6 children) and parent training	Therapists worked with children to address individual needs. Parent training topics which were prioritised according to individual interests and needs.
Functional communication				
**Building Blocks program—home based** [44]	Therapists visited family home to implement intervention with the child, and work with parent(s) to develop skills in working with their child. Focusing on play and natural routines, therapist model skills, give constructive feedback, and discuss issues immediate to the needs of the family. Therapist visits to the pre-school/day-care to observe the child and provide strategies to staff to support skill generalisation.	*Therapists*: teachers, speech pathologists, occupational therapists, psychologists	*40 hours*: 1 x 2-hour home visit/fortnight for 20 fortnights. *Home*: one-on-one therapy	Programs individualised following consultations with parents and other professionals involved in the child’s program.
Functional communication				
**SENSE Theater** [45]	Therapists and peer actors attended 2 days of training in intervention. SENSE Theatre program is manualised. Sessions initially comprised of theatrical games and role- playing exercises. A 45-minute play was introduced in session 3, and participants rehearsed their roles with their peers (learning lines, songs and choreography, character development) for the remaining 7 weeks. Video footage of target behaviours, role-plays and songs acted out by peers viewed by participants as homework. Two public performances of the play given at the end of intervention period.	*Peer*: Typically developing (TD) child of same gender and similar age to participant. *Therapist*: Qualifications not specified	*40 hours in clinic; 17*.*5 hours at home*: 1 x 4-hour clinic sessions/week for 10 weeks; 1 x 15-minute home practice sessions/day for 10 weeks. *Clinic*: group therapy (17 children with ASD, 23 TD peers); *Home*: home practice	Roles in the play were assigned based on individual factors such as age, verbal ability, interests, and talents.
Engage in directed communication				
Use gestures and nonverbal communication in directed ways				
Empathic responding				
**S.S.GRIN-HFA** [46]	Therapists facilitated therapy sessions with participants using a combination of didactic instruction and active practice (e.g. role-play, hands-on activities). Session content divided into 3 modules (5 sessions per module) covering communication, working with others and friendship skills. Parents attended sessions 1, 5, 10 and 15, facilitated home practice, and supported the participant in community based activities.	*Therapist*: Trained in S.S.GRIN-HFA by program developers. Qualifications not specified. *Parent*	*15 hours + home practice*: 1 x 60-minute clinic session/week for 15 weeks; Time for home and community practice not specified. *Clinic*: Group therapy (2 therapists, 27 children); *Home*: Community based practice	None described.
Non-verbal communication				
Listening skills to effectively facilitate conversation				
**FindMe App** [47]	Parents provided with iPad and written instructions dealing with working the iPad and basic troubleshooting. Children used iPad app at home under the guidance of their parents. Activities comprised two parts: Part 1) child identifies the person on the screen; Part 2) child identifies the object that the character on the screen is attending to by following the character’s eye gaze and pointing.	*Parent*	*30–40 hours*: 1 x 5 minute iPad session/day for 6 months or; 3–4 x 10 minute iPad sessions/ week for 6 months. *Home*: iPad App	Levels in the app increased in complexity as children progressed: Part 1) more distractors on screen, some that move; Part 2) character moved to looking only
Attending to people				
Following social cues				
**Therapeutic horse riding** [48]	Lessons comprised two parts: 1) therapeutic riding skills; 2) horsemanship skills. A consistent lesson routine followed: put on riding helmet, wait on bench, mount horse, riding activities, dismount horse, groom horse, and put away equipment.	*Therapist* Certified therapeutic riding instructor	*7*.*5 hours*: 10 x 45 minute sessions/ week for 10 weeks. *Clinic*: Therapeutic horse riding groups (2–4 children and 1 volunteer per child)	None described.
Joint attention				
Nonverbal communication				
**FaceSay** [49]	Therapists trained children in the use of computer hardware and FaceSay computer program for 2 sessions, then facilitated children’s use of the program. Three games from FaceSay program used: 1) Amazing Gazing: touch object on the screen that an avatar is looking at; 2) Band Aid Clinic: select the “band aid” that would fit over the distorted part of an avatar’s face to make it whole; 3) Follow the Leader: identify whether two facial expressions are the same or different.	*Therapist*: Investigators; qualifications not specified	*2–5 hours*: 2 x 10–25 minute sessions/week for 6 weeks; *School*: Computer program	Levels in games increased in complexity as children progressed: more distractors on screen, child is asked to manipulate facial expressions to match a target.
Responding to joint attention				
**JA Intervention (JASPER)** [53, 54]	Therapists were trained in manualised intervention techniques prior to commencement. Sessions began with 5–8 minutes of discrete trial training to prime for target treatment goal at a table. Therapist then used prompting and reinforcement in naturally occurring opportunities to shape targeted skill during semi-structured floor session.	*Therapist*: educational psychology students	*12*.*5–15 hours*: 1 x 30-minute clinic session/day for 5–6 weeks. *Clinic*: One-on-one therapy	Individual child goals determined by outcomes of ESCS, Structured Play Assessment and parent-child interaction. Mastery of goals reached when child demonstrated the goal in 3 different ways at least 3 times at the table and on the floor.
Initiations of joint attention (point, show, give)				
Response to joint attention				
**Modified JASPER Intervention—Parent-child dyad focused** [55]	Therapists facilitated intervention sessions with parent-child dyads using play routines. Session structure: Part 1) 30 mins of direct instruction, modelling, guided practice, and feedback by therapist; Part 2) 10 mins of caregiver practicing techniques learnt. Handouts for caregivers summarizing intervention objectives.	*Therapist*: educational psychology students	*18 hours*: 3 x 45-minute clinic sessions/week for 8 weeks. *Clinic*: One-on-one therapy	Beginning point and modules individualised and determined by interaction in initial parent-child session.
Initiating joint engagement				
Initiating communication				
**Modified JASPER Intervention—Teacher delivered** [51, 52]	A modification of previously manualised treatment (see JASPER [53]). Therapists attended workshop and 5 rehearsal seminars to learn intervention techniques. Therapists then trained teachers in intervention techniques. Teachers facilitated sessions with participants and therapists provided weekly supervision to teachers. Sessions structure: Part 1) 5 mins adult-lead priming for the targeted JA-skill, via toy presentation, prompting, exaggeration of shared interest; Part 2) 15 mins child-lead floor play focusing on generalisation by following the child’s lead, creating play routines, talking about what the child was doing, prompts and responses to JA-skills.	*Teacher; Therapist*: Child and Adolescent Mental Health Clinic counsellors (no qualifications described)	*27 hours*: 2 x 20 minute sessions/day for 8 weeks. *School*: One-on-one therapy	None described.
Initiation of Joint attention (point, show give)				
**JASPER—Caregiver Mediated Model** [56]	Therapists followed manualised intervention aiming to establish didactic engagement between child and caregiver during three home routines (play and two other every day activities). Therapists coached parents in setting up the learning environment, modelling and prompting for joint attention, expanding play and using developmentally appropriate language. A new strategy introduced each week. Handouts provided to parents each week.	*Therapist*: Qualifications not specified	*24 hours*: 2 x 1-hour home sessions/week for 12 weeks. *Home*: One-on-one therapy	None described.
Joint engagement with caregiver				
**JASPER—Caregiver Education Model** [56]	Parents attended training in manualised intervention. Material covered similar to Caregiver Mediated Model (see Kasari, 2014 above) with a focus on behaviour management, developing routines and teaching communication. Weekly handouts provided to parents.	*Therapist*: Qualifications not specified	*24 hours*: 1 x 2 hours session/week for 12 weeks. *Clinic*: Parent group training	None described.
Joint engagement with caregiver				
**Improvisational music therapy** [57]	Semi-flexible treatment manual developed. Instruments available included piano, cymbals, drums, xylophone, harp, bells and shakers, horns and whistles. Session structure: Part 1) 15 mins undirected child-led activity with therapist supporting and elaborating on child’s play; Part 2) 15 mins directed activity with therapist modelling turn-taking activities within child’s focus and interest.	*Therapist*: music therapists, play therapists, music therapy students	*6 hours*: 1 x 30 minute sessions/week for 12 weeks. *Clinic*: One-on-one therapy	None described.
Joint attention behaviours (eye-contact, turn taking)				
**SummerMAX + Mind Reading (MR) computer program** [39]	See Skillstreaming for description of session structure and parent component. MR was implemented in addition to Skillstreaming 3 sessions per week, and replaced the emotion recognition instruction typically implemented in Skillstreaming.	*Therapist*: Graduate and undergraduate students (discipline not specified)	*145 hours for child; 7*.*5 hours for parent*: 5 x 70 minute treatment cycles, 5 days per week, for 5 weeks; 1 x 90 minute parent training sessions/ week for 5 weeks. *Clinic*: Group therapy (6 children and 3 therapists)	See Skillstreaming and MR
Social-communication				
Face-emotion recognition				
**Skillstreaming** [38]	Manualised intervention. Session structure: Part 1) 20 mins instruction in target skill; 2) 50 mins therapeutic activity. To conclude, each child discussed the social skills they used to complete the activity. Activities provided practice in and reinforced identifying and interpreting idioms, multiple meanings of common language, identifying facial features, positions and physiological reactions that characterise different emotions. Skills targeted via direct instruction, modelling, role-playing, feedback, and transfer of learning. A concurrent parent training group focused on increasing understanding of autism and the intervention techniques.	*Therapist*: education and psychology students	*145 hours for child; 7*.*5 hours for parent*: 5 x 70 minute treatment cycles, 5 days per week, for 5 weeks; 1 x 90 minute parent training sessions/ week for 5 weeks. *Clinic*: Group therapy (6 children and 3 therapists) and parent training.	Social skills were taught in a progression from basic to more complex. The same skills were taught to all participants; however, skills were tailored to participant age so that target skills reflected of social situations/demands encountered by children of various ages. Progression of face-emotion recognition activities: 1) identification of facial expressions in pictures; 2) examination of other children’s expressions during activities; 3) identification of physiological reactions associated with different facial expressions. Individualised daily contract of 2–3 targets not covered in the curriculum.
Face-emotion recognition				
**Emotion recognition training** [58]	Therapists facilitated intervention sessions. Sessions comprised of direct instruction on components of six target facial expressions, and practice opportunities (e.g. role play, drawing, matching games). Workbooks completed as homework following sessions 1–3. Parents encouraged to assist with homework and attend information evening on therapeutic techniques.	*Therapist*: Qualifications not specified	*4 hours*: 1 x 1-hour clinic sessions/week for 4 weeks. *Clinic*: one-on-one therapy; *Home*: home practice	None described.
Emotion recognition through facial expression				
**Seaver-NETT** [59]	Treatment facilitated manualised intervention. Session structure: Part 1) 15 mins free-play/snack time; Part 2) 60 mins instruction; part 3) 15 min wrap-up time. Instruction followed a modular cognitive behavioural intervention-based curriculum via didactic instruction, reinforcement activities, visual supports, skills practice and a token economy for reinforcement. Parent training ran concurrently with group therapy sessions, covering treatment rational, homework review and discussion.	*Therapist*: Clinical psychologist and therapy assistants	*18 hours for child; 6 hours for parent*: 1 x 90-minute clinic session/week for 12 weeks; 1 x 30-minute parent sessions/week for 12 weeks. *Clinic*: group therapy (4–6 children, 2–3 interventionists) and parent training	Consideration for individualisation described in reference to manual content, but procedures not specified.
Nonverbal communication				
Emotion recognition				
**Mind Reading (MR) computer program** [40]	Therapists attended 8 hours of training in intervention protocol, and were required to pass exam. Session structure: 1) MR training: audio-visual stimuli of voices and faces teach children to recognise 412 simple and complex emotions through observation of emotion expressions, structured lessons, quizzes, “games” for additional practice, and rewards; 2) in vivo rehearsal trials presented at 5 intervals during sessions provide additional practice at decoding and encoding target expressions ono-on-one with a therapist; 3) a “points” system to rewarded behaviour and decoding/encoding skills.	*Therapist*: education and psychology students	*36 hours*: 2 x 90-minute clinic sessions/week for 12 weeks. *Clinic*: computer program, one-on-one in vivo rehearsal trials.	None described.
Facial expression decoding				
Prosody decoding				

**Table 4:** Pragmatic language skills targeted by included interventions.

Intervention	Pragmatic language skills					
	Preverbal pragmatic language	Introduction and responsiveness	Nonverbal comm.	Social Emotional attunement	Executive Function	Negotiation
TJDP [41]		♦	♦			
MEHRI Treatment [42, 43]	♦	♦		♦		
SENSE Theater [45]		♦	♦	♦		
S.S.GRIN-HFA [46]		♦	♦	♦		
FindMe App [47]	♦		♦			
Therapeutic Horse-riding [48]	♦		♦			
FaceSay [49]	♦	♦				
JA Intervention (JASPER) [53, 54]	♦	♦	♦			
Modified JASPER Intervention—Parent-child dyad focused [55]	♦	♦	♦			
Modified JASPER Intervention—Teacher delivered [51, 52]	♦	♦	♦			
JASPER—Caregiver Mediated Model [56]	♦			♦		
JASPER—Caregiver Education Model [56]	♦			♦		
Improvisational music therapy [57]	♦		♦	♦		
SummerMAX + MR [39]			♦			
Skillstreaming [38]			♦			
Building Blocks program—center based [44]		♦				
Building Blocks program—home based [44]		♦				
Emotion recognition training [58]			♦			
Seaver-NETT [59]			♦			
Mind Reading (MR) computer program [40]			♦			

**Table 5:** Methodological quality of included studies.

Study	Treatment	Control	Randomisation	Blinding	Methodological Quality
Beaumont and Sofronoff [41]	**TJDP:** Child focused, computer based activities with small group therapy	Wait-listed control	Randomisation reported but procedure not described.	No blinding of participants or investigators reported.	Good quality: 75%
Casenhiser, Shanker [42]	**MEHRI Treatment:** Parent and child focused, social-interaction based therapy	Community treatment control	Randomisation stratified by age, language and cognition level at entry. Random number generator used to assign participants to groups following screening.	No blinding of participants or therapists reported. Coding of interactions completed by independent coders who were blind to intervention group. Blinding to testing time not reported.	Good quality: 75%
Casenhiser, Binns [43]	**MEHRI Treatment:** Parent and child focused, social-interaction based therapy	Community treatment control	Randomisation stratified by age, language and cognition level at entry. Random number generator used to assign participants to groups following screening.	No blinding of participants or therapists reported. Coding of communication acts completed by independent coders, with videos numbered to disguise group assignment and testing time.	Adequate quality: 64%
Corbett, Key [45]	**SENSE Theater:** Child focused, theatre based group therapy	Wait-listed control	Randomisation reported but procedure not described.	No blinding of participants or investigators reported.	Adequate quality: 61%
DeRosier, Swick [46]	**S.S.GRIN-HFA:** Child focused, cognitive behavioural and social learning based group therapy adapted to focus on social challenges pertinent in ASD	Child focused, cognitive behavioural and social learning based group therapy	Randomisation reported but procedure not described.	No blinding of participants or investigators reported.	Adequate quality: 57%
Fletcher-Watson, Petrou [47]	**FindMe App:** Child focused iPad app	Treatment as usual control	Randomisation stratified by ADOS social communication score. Block randomisation with varying and randomly ordered block size produced by independent researcher.	Baseline assessments administered and scored by first author prior to group allocation. Participants and investigators not blinded to group allocation. Post-assessments were parent report measures and therefore not blinded. Coding of videos for follow-up assessment completed by independent rater, blind to group allocation.	Adequate quality: 68%
Gabriels, Pan [48]	**Therapeutic Horse-riding:** Child focused, therapeutic horse riding intervention	Barn activities	Stratified by nonverbal IQ by project’s statistician using size 4 block randomisation.	Ratings of social communication completed by caregiver and therefore unblinded. Blinding of therapists and participants not reported.	Adequate quality: 64%
Hopkins, Gower [49]	**FaceSay:** Child focused, computer based intervention	Computer based drawing program	Randomisation reported but procedure not described.	No blinding of participants or investigators reported for pragmatic language outcome measures.	Poor quality: 46%
Kaale, Smith [51]	**Modified JASPER Intervention—Teacher delivered:** Teacher delivered, child focused, joint attention intervention	Ordinary pre-school program	Randomisation conducted by the first author following baseline assessment. The list, generated by an independent statistician, contained random blocks of four for each study site and was not stratified. The list was generated so as to ensure equal distribution of participants to both the intervention and control group at each recruitment site.	Participants and investigators blind to treatment group at baseline assessment. Video coding for social communication outcomes completed by research assistants blinded to study purpose, group allocation and testing time.	Good quality: 79%
Kaale, Fagerland [52]	**Modified JASPER Intervention—Teacher delivered:** Teacher delivered, child focused, joint attention intervention	Ordinary pre-school program	Randomisation reported but procedure not described.	Blinding of participants and therapists not reported. Video coding for social communication outcomes completed by research assistants blinded to study purpose, group allocation and testing time. All other assessments administered by independent researchers, blind to group allocation.	Good quality: 79%
Kasari, Freeman [53]	**JA Intervention (JASPER):** Therapist delivered, child focused, joint attention intervention	Treatment as usual control	Randomisation of participants to groups reported but procedure not described. Randomisation of therapists to treatment procedure and child reported but procedure not described.	Blinding of participants and therapists not reported. Staff in the intervention setting were independent of the research staff and blind to the study hypotheses. Video coding for social communication outcomes completed by independent coders blinded to group allocation. Screening assessments administered by independent researchers, blind to study purpose and hypotheses.	Adequate quality: 61%
Lawton and Kasari [54]	**JA Intervention (JASPER):** Therapist delivered, child focused, joint attention intervention	Treatment as usual control	Randomisation of participants to groups reported but procedure not described.	Blinding of participants, therapists and video coders not reported. Screening assessments administered by independent researchers, blind to group allocation.	Adequate quality: 57%
Kasari, Gulsrud [55]	**Modified JASPER Intervention—Parent-child dyad focused:** Therapist delivered, parent focused, joint attention intervention	Wait-listed control	Random numbers method used to randomise participants to condition.	Blinding of participants and therapists not reported. Video coding for social communication outcomes completed by independent coders blinded to group allocation and testing time.	Good quality: 71%
Kasari, Lawton [56]	**JASPER—Caregiver Education Model:** Caregiver group training, joint attention intervention	Treatment as usual	Randomisation of participants to groups conducted by independent data centre, but procedure not described.	Blinding of participants and therapists not reported. Assessments administered at all time points by examiners blind to treatment condition and study hypotheses. Analysis conducted by independent data centre. Video coding for social communication outcomes completed by independent coders blinded to group allocation.	Adequate quality: 68%
Kim, Wigram [57]	**Improvisational music therapy:** Therapist delivered, child focused, improvisational music therapy	Play sessions	Randomisation of participants to groups reported but procedure not described.	Blinding of participants and therapists not reported. Video coding for social communication outcomes completed by independent coders blinded to treatment condition.	Poor quality: 36%
Lopata, Thomeer [38]	**Skillstreaming:** Therapist delivered, child focused, social skills group therapy	Wait-listed control	Randomisation stratified on age, gender and ethnicity. One researcher randomly assigned numbers to participants, and a second researcher used a table of random numbers to assign numerically identified children	No blinding of participants, therapist or testers reported.	Good quality: 75%
Lopata, Thomeer [39]	**SummerMAX + MR:** Therapist and computer delivered, child focused, group therapy	Therapist delivered, child focused, group therapy	Randomisation of participants to groups conducted using an online random number generator	Researchers and participants unaware of treatment allocation at baseline assessment. Post-assessments conducted by researchers blind to study hypothesis. Binding of therapists not reported.	Strong quality: 82%
Roberts, Williams [44]	**Building Blocks program—home based:** therapist delivered, child and parent focused, home based therapy.	Wait-listed control	Randomisation completed using computer generated random number tables.	No blinding of participants, therapist or testers reported.	Adequate quality: 68%
	**Building Blocks program—center based:** Therapist delivered playgroup and parent training.				
Ryan and Charragain [58]	**Emotion recognition training:** Therapist delivered, child focused, emotion recognition therapy.	Wait-listed control	Randomisation of participants to groups reported but procedure not described.	Blinding of participants and therapists not reported. Post-measures administered by psychologist who was blinded to pre-scores. Not reported whether tester was blind to treatment allocations as well.	Adequate quality: 57%
Soorya, Siper [59]	**Seaver-NETT:** Therapist delivered, child focused, group intervention with concurrent parent training.	Facilitated play sessions	Participants randomised by computer generated randomisation in blocks of 10–12 over 7 recruitment phases	Ratings of social communication completed by caregiver and therefore unblinded. Blinding of therapists and participants not reported.	Good quality: 75%
Thomeer, Smith [40]	**Mind Reading (MR) computer program:** Child focused, computer based intervention with in vivo rehearsal trials.	Wait-listed control	Participants randomised to groups using online number generator.	Blinding of participants and therapists not reported. No description provided as to who administered primary measurements of social communication, and no report of tester blinding. Secondary measurement of social communication was completed by parents via questionnaire, and parents could not be blinded to treatment condition.	Good quality: 68%

#### Study participants

The 21 studies that met the eligibility criteria included 925 participants aged between 21 months and 14 years of age. Of the 21 included studies, 11 studies included preschool aged children (younger than 5 years), and 10 studies included primary/elementary school aged children (aged between 5 and 12 years inclusive). None of the included studies targeted children aged 13–18 years.

All intervention and control group participants had received a diagnosis of ASD in accordance with the DSM-IV or DSM-5 prior to being included in all studies. No study included control groups from different clinical populations or typically developing children. ASD diagnosis was confirmed in 20 studies by administering standardised assessments of ASD symptomology to participants, and one study confirmed diagnosis via diagnostic documentation from qualified community clinicians [38]. The absence of an intellectual disability or another neurological or developmental disability was a criterion for inclusion for 12 studies. Of these 12 studies, nine assessed cognitive capacity for inclusion using a standardised assessment appropriate for the age of the included participants, and the remaining three utilised parent report as the children were too young to undertake formal IQ testing (i.e., under 6 years of age). Three studies required that participants demonstrate age appropriate expressive or receptive language prior to inclusion [38–40]. Treatment group sample sizes ranged from five to 59, with nine of the papers reporting calculations of power to determine an appropriate sample size. Further details on participant characteristics are summarised in Table 2.

#### Outcome measures

The method of outcome data collection varied across the 21 papers. Behavioural observation was the most common method of pragmatic language skill measurement, with 11 reports utilising this approach. Behavioural observations typically involved recording the child interacting in a social context (e.g., playing with a parent, interacting in the playground), and coding the footage for pragmatic language behaviours of interest. Parent report measures were administered in six studies. These measures required parents to complete a standardised questionnaire about their child’s social communication competence. One study utilised both observational and parent report measures [47]. Standardised lab tasks assessing emotion recognition were administered to study participants in five studies. Specific assessments and methods for collection are described in Table 2.

Pragmatic language skills measured by these assessments varied greatly across studies. Of the 11 papers that included behavioural observations, eight studies collected data pertaining to initiations of joint attention, three measured joint engagement, three measured responsiveness to another’s communicative attempts, one measured verbal initiations, one measured frequency of requests, and one coded communicative acts. The five studies that administered assessments directly to participants all measured emotion recognition via non-verbal cues such as facial expression, posture, gesture or prosody. All parent report surveys measured capacity for reciprocal social communication.

#### Results reported

Pre-post data were reported in 20 papers, with Kaale, Fagerland [52] reporting on the 12-month follow-up data from the study originally reported by Kaale, Smith [51]. Follow-up data were presented in nine papers, with time frames ranging from 5-weeks to 12-months post cessation of intervention. Lawton and Kasari [54] reported on results collected from the same sample following the same course of intervention as Kasari, Freeman [53], but using an alternative outcome measure at four time points: pre, post, 6-month follow-up and 12-month follow-up. Casenhiser, Shanker [42] and Casenhiser, Binns [43] also reported results from the same intervention study, with the latter presenting a re-analysis of the video data collected for an alternative purpose. The treatment outcome(s) for each study is presented in Table 3.

#### Interventions

A detailed description of each intervention is provided in Table 3. Twenty different intervention programs were reported across the 21 studies, although four were various modifications of the Joint Attention, Symbolic Play and Engagement Regulation [JASPER] intervention initially reported by Kasari, Freeman [53]. Originally a clinic based, therapist facilitated, individual, child-focused intervention for joint attention skills, JASPER approach was first modified to include a focus on the parent-child dyad [55]. It was later trialed as a teacher delivered, school-based intervention [51, 52]. Most recently JASPER was implemented via two models of parent delivered intervention: 1) Caregiver Mediated Model (CMM); and 2) Caregiver Education Model (CEM) [56]. Education of the parent was the focus of these approaches, with CMM being delivered by the therapist to both the child and parent in a one-on-one setting at home, and CEM delivered in a group setting with parents only. Additionally, Lopata, Thomeer [39] studied a treatment protocol which combines the intervention approaches reported on by Lopata, Thomeer [38] and Thomeer, Smith [40].

The mode of delivery and focus subject of the interventions varied across the studies. Pragmatic language skills were targeted in a group setting in nine intervention protocols. Of those nine approaches, five were child directed interventions, one focused on educating parents [56], and three focused on both the children and parents. An individual approach to intervention was taken in 11 studies, of which seven were child focused. The remaining four individual interventions focused on the child and the parent through direct intervention of the therapist with the child, along with training parents in therapeutic techniques to support their child. A combination of group and individual activities were employed in two interventions and both of these focused on the children only [41, 59].

Clinics were the setting for 15 of the interventions, and five of these also included out of session practice either at home or in the community. All clinic based interventions were facilitated by a therapist trained in the particular intervention program, with one also utilising the parent as an interventionist while completing computer based activities [41], and one including the use of typically developing peers in the group intervention [45]. Three interventions were implemented in the child’s home and these were all facilitated by a trained therapist. The child’s school was the setting for two interventions, with one being a therapist facilitated computer based intervention [49] and the other being facilitated by teachers who were trained in the intervention procedures by therapists [51, 52].

Interventions varied in frequency (i.e., the number of times the intervention is provided per day or per week) and total intervention duration (i.e., the time period over which the intervention is presented). The shortest intervention was the Emotion Recognition Intervention [58] which was conducted over four weeks; totalling four hours of intervention. The longest intervention was the MEHRI treatment [42, 43] implemented over 12 months, totalling 104 clinic hours and 1,092 home-based hours. Eight of the interventions had a total duration of 10–15 weeks, with the most frequently occurring duration being 12 weeks. Eight interventions were implemented in fewer than 10 weeks, and four interventions lasted 26 weeks or more. The intervention with the lowest intensity was the improvisational music therapy [57], which required 30 minutes of intervention per week. The most intense intervention was Skillstreatming and SummerMAX + Mind Reading which involved five daily 70-minute treatment “cycles”, five days per week for five weeks, equating to 29 intervention hours per week [39]. The most common session frequency was weekly, with 11 interventions running weekly sessions with the interventionist. Only two studies reported an expected frequency for home-practice between sessions, and both interventions required daily practice. Five interventions ran on at least a daily basis, with a modified JASPER intervention occurring twice daily [51, 52] and Skillstreatming and SummerMAX + Mind occurring five times daily [38]. The least frequently occurring intervention sessions occurred in the Building Blocks program—home based [44], with the clinician visiting the participant’s home every other week; no specific practice between sessions were described.

A synthesis of the pragmatic language skills targeted by each intervention is provided in Table 4. The most frequently targeted skill was nonverbal communication with 14 interventions focusing on the use and interpretation of gesture, facial expressions and/or tone of voice. Introduction and responsiveness was the target of 10 interventions, 10 interventions also targeted preverbal social communication behaviours, and 4 interventions targeted social emotional attunement. No one intervention reported targeting all pragmatic language skills adopted for this review, and no intervention targeted the skills of executive function or negotiation.

#### Control groups

All participants included in control groups had a diagnosis of ASD. Seven studies assigned control participants to waitlisted control groups who served as a no-treatment comparison during the intervention phase of the project then went on the receive the intervention at a later stage. Control participants in five studies attended clinic sessions at the same frequency as the intervention group, but participated in activities that were hypothesised not to treat the targeted skill set (e.g., computer based drawing activity, facilitated play with toys). Control groups in nine studies were assigned to a treatment as usual group where the “usual treatment” reflected typical intervention practice in the setting in which the study was set (e.g., typical preschool program, an alternative social skills program with differing intervention practices [46, 51]).

#### Methodological quality

A description of the methodological quality and Kmet ratings of the included studies is provided in Table 5. One study, reporting on the effectiveness of SummerMAX + Mind Reading [39], was rated as having strong methodological quality using the Kmet checklist. Good methodological quality was measured in 8 of the papers. One of these reported on results of The Junior Detective Program [41], one reported on the MEHRI treatment [42], three reported on different adaptations of JASPER [51, 52, 55], one reported on Skillstreaming [38], one reported on the Seaver-NETT program [59], and one reported on the Mind Reading computer program [40]. Adequate methodological quality was rated in 9 papers, and the remaining 2 were rated as having poor methodological quality.

#### Risk of bias in studies

All studies reported randomisation of participants to groups, and 10 detailed the procedures for random allocation in detail. The remaining 11 studies did not report on the generation of the allocation of participants to groups and so the risk of bias in these studies is unclear. All included studies were at risk of bias due to challenges in blinding of participants, their families and those involved in administering the interventions; an acknowledged difficulty in designing clinical intervention research [61]. However, blinding of outcome measurements was reported in eight studies that utilised observational measures of pragmatic language [42, 43, 51–53, 55–57]. In these studies, video recorded observations were coded and rated by independent researchers unaware of the participants’ group allocation or time in the study when the observations were collected. Raters in three of the studies were also blind to the purpose of the study [51–53]. Two further studies reported observational measures of pragmatic language, but it is not clear whether observers were blinded [49, 54]. The risk of bias in the outcome measurements of all other studies is either evident or unknown. The researchers either administered assessments directly to the child, or collected information via parent survey and are at risk of bias due to unclear reports of blinding for child directed assessments, and an inability to blind parent-rated outcome measurements.

Sample size calculations were reported and an appropriate sample size was used in 9 studies, leaving the risk of bias unclear in the remaining 12 studies. A potential invested interest bias was apparent in a number of studies, with authors having conducted previous research on the same topic, or being involved in the development of the intervention protocol being investigated [38–41, 53–56].

The fail-safe N calculated during meta-analysis was 108, meaning as many nil effect studies would need to have been conducted and not published in order to negate the observed effect of the included studies. Such a large N-value indicates a low risk of publication bias.

### Effects of interventions: Meta-analysis results

Fifteen of the 21 studies were included in the meta-analysis. Three studies [40, 46, 57] could not be included in the analysis as the data required for calculations were not reported. The authors were contacted to collect the required data needed for the meta-analysis, but none of the authors responded to the requests. A further two studies were excluded [42, 53], as they reported on the same sample as two other studies [43, 54], but used outcome measures that evaluated a narrower range of pragmatic language skills. One final study was excluded as it reported on 12-month follow up data only [52]. Seven studies measured social communication using more than one instrument. A single outcome measure was extracted for inclusion in the analysis from four of these studies, as the measure chosen was likely to reflect a more comprehensive suite of pragmatic language skill than the others reported [39, 54–56]. The remaining three articles reported two or more similar measurements of a single pragmatic language construct [41, 43, 51], so the mean scores were averaged and pooled standard deviations were calculated for each study for use in the analysis. There were 17 participant samples across the 15 included studies, as two studies contained two intervention groups [44, 47].

Overall treatment effects were calculated for pragmatic language interventions on pre-post outcome measures. Sub-group analysis was conducted to compare the effect as a function of three intervention characteristics: 1) setting (i.e., clinic, home, school), intervention focus (i.e., child focused, parent focused, or both), and mode of delivery (i.e., group interventions, one-on-one interventions or both). Further analysis was conducted to detect whether participant age, outcome measure type, intervention setting, focus or mode of delivery mediated intervention effect. Between groups analysis was also conducted to compare post-intervention scores with control groups, grouped by control condition type. Three control condition types were included: 1) waitlisted control groups where participants served as an untreated comparison group who eventually went on to receive the intervention; 2) treatment as usual control groups where participants received interventions typically prescribed in the clinic or school in which the intervention was set; and 3) alternative treatment controls where participants attended the clinical setting but participated in an activity that reflected the intervention approach without the activity that was thought to be the agent of change.

#### Overall effect of pragmatic language interventions

**Fig 2:**
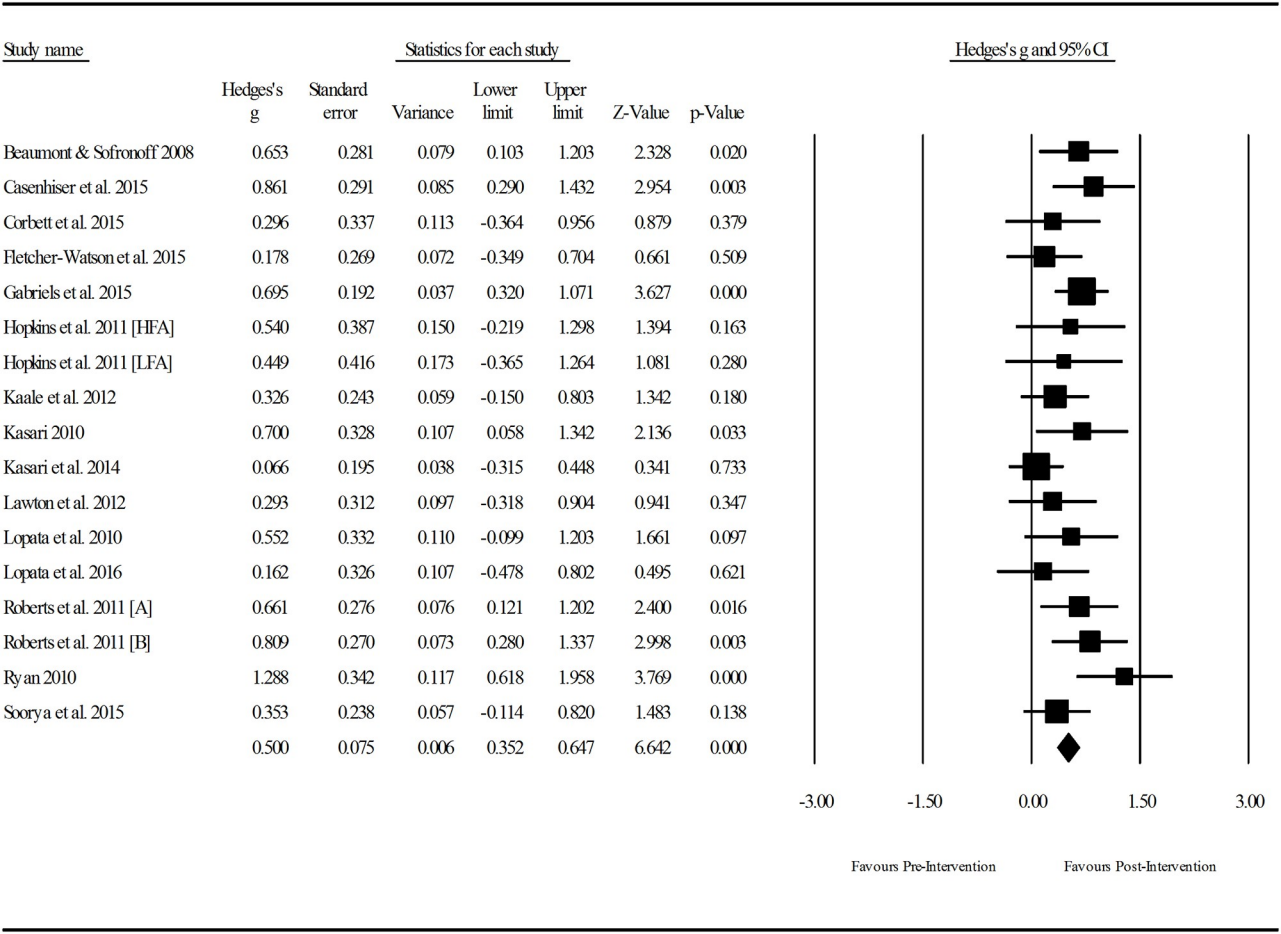
Within intervention group pre-post meta-analysis. *Notes*: Hedge’s *g* interpreted as per Cohen’s *d* conventions: ≤0.2 = negligible difference, 0.2–0.49 = small, 0.5–0.79 = moderate, ≥ 0.8 = large.

Effect sizes ranged from 0.162 to 1.288 in the pre-post intervention within groups analysis, as shown in Fig 2. Of the 17 intervention groups sampled, 24% produced a large effect, 29% proceed a medium effect, and 29% produced a small effect. An effect size < 0.2 was measured in 18% of the intervention groups. A small but significant post-intervention between-groups total effect size was found, favouring pragmatic language interventions for children with ASD (*z*(17) = 2.889, *p* = 0.004, Hedge’s *g* = 0.274, 95%CI = 0.088–0.460). The overall intervention effect was moderate (*z*(17) = 6.642, *p* < 0.001, Hedge’s *g* = 0.500, 95%CI = 0.352–0.647). The between-study heterogeneity was not significant (*Q*(16) = 19.413, *p* = 0.248), and 17.570% of true variability (*I*^*2*^) could be explained by individual study characteristics. Following the subgroup analysis of intervention characteristics meta-regression analysis was performed to further explain variability in the results.

#### Effect size as a function of intervention characteristics

**Fig 3:**
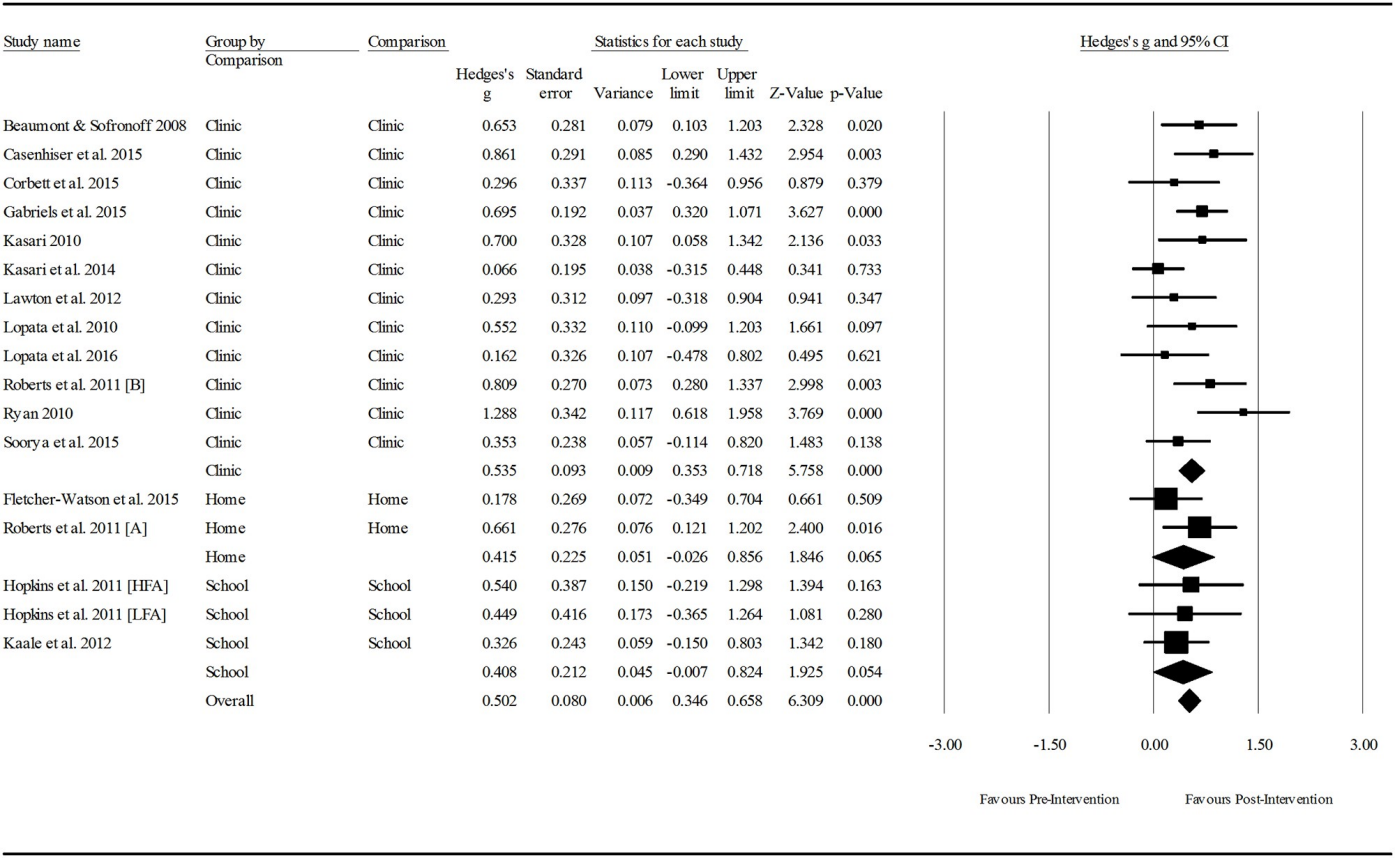
Within intervention group pre- post- meta-analysis, grouped by setting. *Notes*: Hedge’s *g* interpreted as per Cohen’s *d* conventions: ≤0.2 = negligible difference, 0.2–0.49 = small, 0.5–0.79 = moderate, ≥ 0.8 = large. Clinic: participants attended the interventionists premises; Home: clinicians visited participant’s home OR parents administered intervention at home; School: intervention was carried out at the participants’ school outside of the normal curriculum.

**Fig 4:**
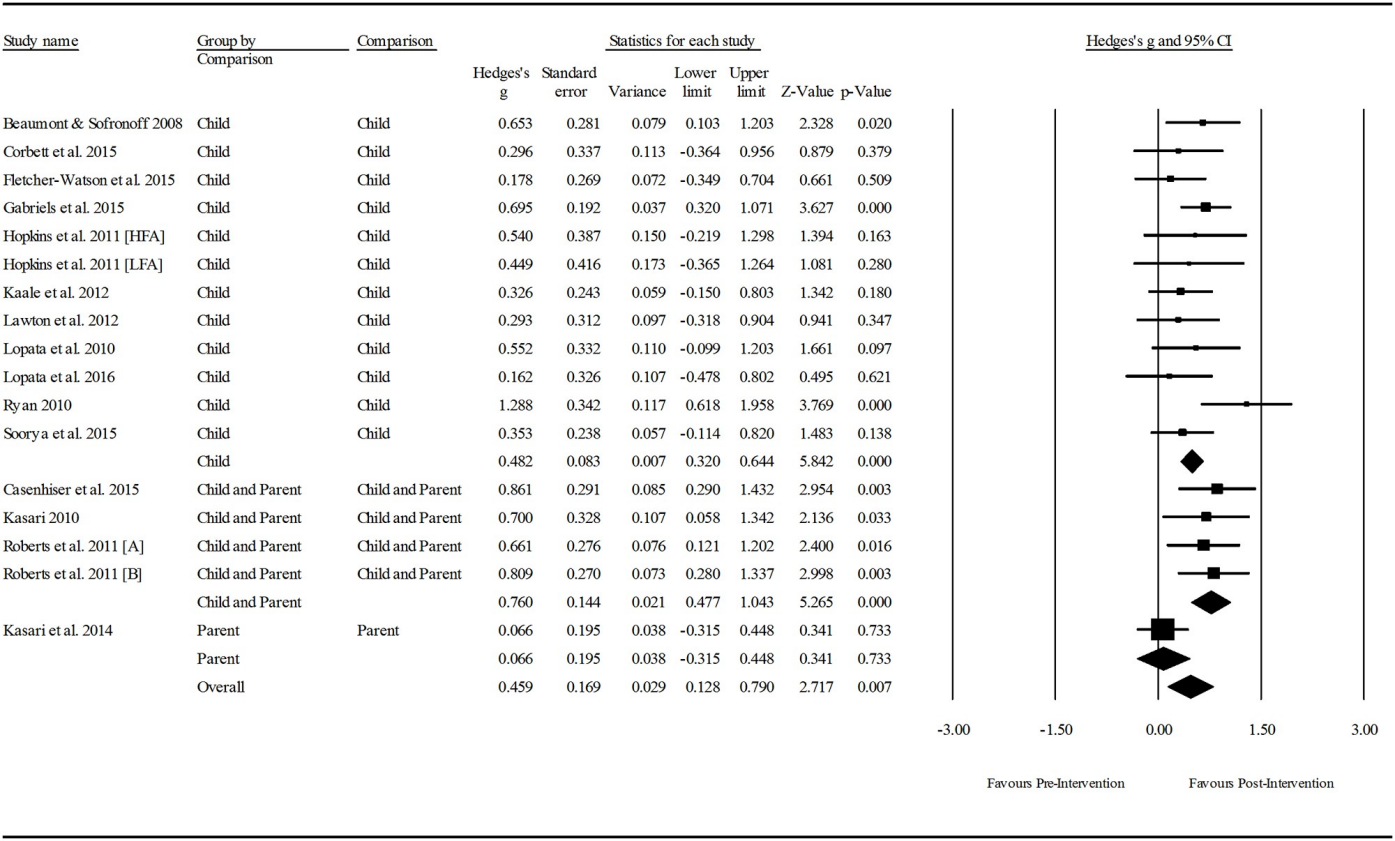
Within intervention group pre- post- intervention meta-analysis, grouped by therapy focus. *Notes*: Hedge’s *g* interpreted as per Cohen’s *d* conventions: ≤0.2 = negligible difference, 0.2–0.49 = small, 0.5–0.79 = moderate, ≥ 0.8 = large. Child: interventions were administered to the participants only either in groups or individually; Child and parents: parent training and//or education were integrated into intervention sessions either concurrently with the child/ren or in separate sessions; Parent: sessions only involved parent education.

**Fig 5:**
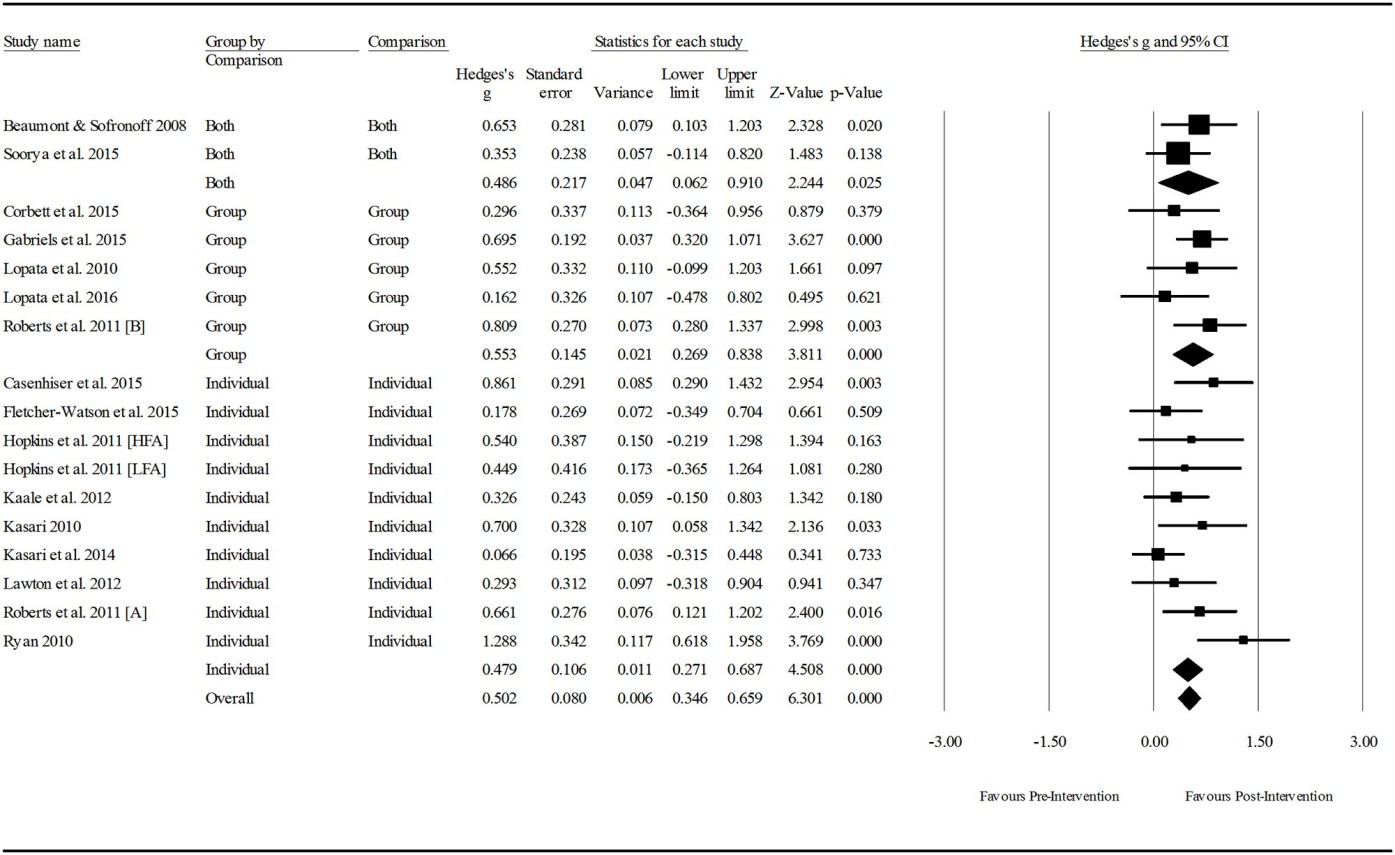
Within intervention group pre- post- treatment meta-analysis, grouped by mode. *Notes*: Hedge’s *g* interpreted as per Cohen’s *d* conventions: ≤0.2 = negligible difference, 0.2–0.49 = small, 0.5–0.79 = moderate, ≥ 0.8 = large. Individual: interventions were administered in a one-on-one setting; Group: interventions were administered to participants in small groups; Both: sessions were comprised of individual and group aspects.

Figs 3 to 5 indicate the effect sizes of pragmatic language interventions grouped by setting, focus and mode of delivery respectively. Interventions set in the clinic demonstrated a significant, moderate effect size (z(12) = 5.758, p < 0.001, Hedge’s g = 0.535, 95%CI = 0.353–0.718), which was the largest effect size calculated as a function of setting. Interventions set in the school were approaching significance, with a small effect (*z*(3) = 1.925, *p* = 0.054, Hedge's *g* = 0.408, 95%CI = -0.007–0.824), and interventions set in the home did not have a significant effect on improving pragmatic language skills when compared to the other settings (*z*(2) = 1.846, *p* = 0.065). However, these results should be interpreted with caution as only two studies were set in the home and just one at school compared to 12 in the clinic setting group. Approaches that integrated a caregiver into the program via education and/or coaching in intervention techniques demonstrated a significant, moderate-large effect (*z*(4) = 5.265, *p* < 0.001, Hedge’s *g* = 0.760, 95%CI = 0.477–1.043), while the intervention that focused on parent education only had no significant impact on the pragmatic language skills of children with ASD (*z*(1) = 0.341, *p* = 0.733). The majority of studies focused on administering the intervention directly to the children with ASD, and these interventions demonstrated a significant, moderate effect (*z*(12) = 5.842, *p* < 0.001, Hedge’s *g* = 0.482, 95%CI = 0.320–0.644). Again, caution is required in interpreting these results as there is only one study in the parent focused group, and 12 and 4 in the child focused and combined child and parent focused groups respectively. Whether interventions were administered to a group, the individual or both, effects were significant and moderate in size. Group interventions produced the largest effect of the three modalities (*z*(5) = 3.811, *p* < 0.001, Hedge’s *g* = 0.553, 95%CI = 0.269–0.838).

#### Factors mediating intervention effect

No differences were detected in outcomes as a result of participant age or method of pragmatic language measurement (i.e., parent report, observation, or lab task). The analysis of intervention characteristics indicated that intervention setting and mode were not significant mediators of intervention effect. However, intervention focus (e.g. child, parent or child and parent) was found to be a significant mediator of pragmatic language outcomes (*F*(2) = 4.17, *p* = 0.0381), accounting for all of the between-study variance in the model (*R*^*2*^ = 100%). Lastly, as there was a concordance between increased age and receiving intervention in a group, participant age was examined in relation to mode. This did not produce a significant result, indicating age did not mediate the effect of mode of delivery (i.e., individual, group, or both).

#### Effect of pragmatic language interventions compared with comparison groups

**Fig 6:**
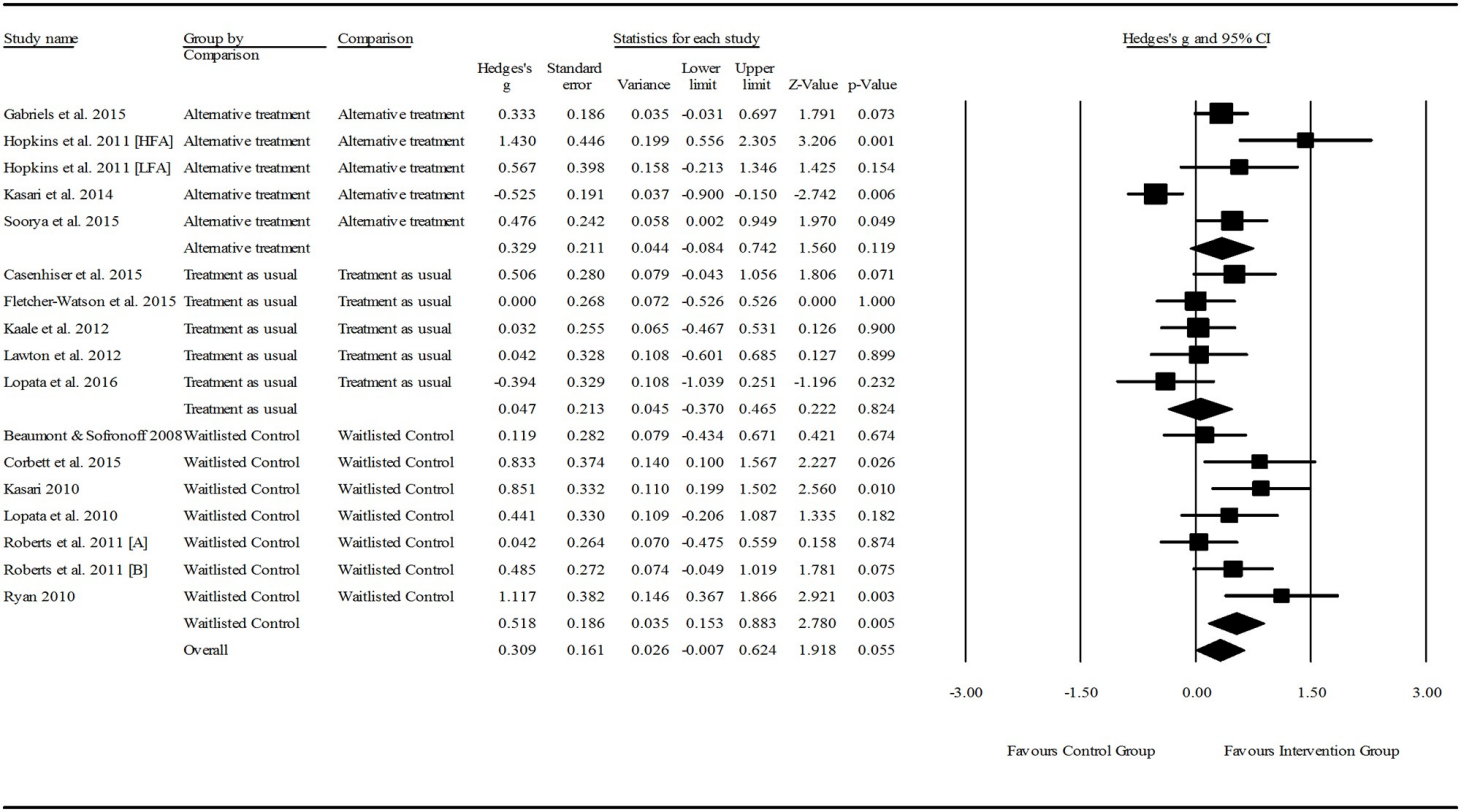
Between intervention groups post-score meta-analysis, grouped by control group type. *Notes*: Hedge’s *g* interpreted as per Cohen’s *d* conventions: ≤0.2 = negligible difference, 0.2–0.49 = small, 0.5–0.79 = moderate, ≥ 0.8 = large. Alternative treatment: control groups attended an activity that reflected aspects of the intervention without the components thought to be crucial in improving pragmatic language; Treatment as usual: control groups received the intervention or education program typically administered in the intervention setting; Waitlisted control: control groups served as an untreated comparison.

As shown in Fig 6, pragmatic language interventions for children with ASD showed a moderate, significant effect when compared to the waitlisted control group (*z*(7) = 2.780, *p* = 0.005, Hedge’s *g* = 0.5.18, 95%CI = 0.153–0.883). Customised pragmatic language interventions did not have a significant effect when compared to an alternative treatment (*z*(5) = 1.560, *p* = 0.119) or treatment as usual (*z*(5) = 0.222, *p* = 0.824). Effect size of intervention compared to waitlisted controls was similar to that of the overall pre-post results for all interventions.

## Discussion

This study aimed to review and analyse the evidence-base for interventions to improve pragmatic language skills in children with ASD. Using procedures as outlined by the PRISMA statement [62], a systematic review and meta-analysis of RCT studies were conducted.

Participants in all 21 included papers were of pre-school or elementary/primary school age. Associations between early intervention for children with ASD and reduced symptom severity in the long term are widely accepted. Similarly, gestural non-verbal joint attention has been shown to be predictive of later language acquisition in children with ASD [5]. As such, providing effective interventions for early developing pragmatic language skills to verbal and minimally verbal pre-school aged children is likely to have a crucial impact on future social and linguistic development. The two interventions producing a large effect on pragmatic language for the 0–5 year age group were clinic-based approaches that focused on developing functional language use [42–44]. Other interventions for this age group targeted giving and responding to non-verbal communication acts to engage in joint attention with a social partner, produced negligible to moderate effect sizes, indicating a need for further development and investigation of these interventions.

Interventions for children aged 6–12 years broadly targeted children without any comorbid language or neurodevelopmental disorders. A similar gap is highlighted in the boarder language and communication intervention literature for minimally verbal children with ASD in this age group [6]. Studies of older children, like those included in this review, focus on verbal children and it is suggested that adapting interventions designed for younger children with ASD could provide potential intervention approaches for older, minimally verbal children with ASD [6]. Given the large effect of interventions such as Building Blocks in targeting pragmatic language in under five year olds [44], adaptations of these approaches may be a viable option for further investigation for minimally verbal older children with ASD. Randomised controlled trials assessing pragmatic language outcomes following the introduction of an alternative support for the production of language (e.g., Picture Exchange Communication System (PECS), or the use of speech production applications/devices), of which this review found none, could also provide future evidence for interventions appropriate to this population.

This review did not find any evidence for any effective pragmatic language interventions for adolescents with ASD, highlighting a gap in the continuity of effective interventions for individuals with ASD as their social environment evolves and becomes more complex. A more multifaceted set of pragmatic language skills is required as children continue to develop from early childhood into adolescence and adulthood. Pragmatic language interventions that recognise the increasing complexity of social interactions would aid in the reduction of the long-term psychosocial impacts that these deficits can have on the development of quality relationships [14], which in turn can reduce social exclusion and promote resilience [63].

Intervention was provided in a group setting in 13 of the studies. At an aggregate level, the group interventions were significantly more effective than individually focused interventions, but by a small magnitude. Interestingly, a majority (80%) of the group-based interventions were also focused on the older age cohort (6–12 years), potentially mediating the sub-group analysis by mode. However, the results of the meta-regression indicate that interventions delivered at different ages resulted in similar outcomes. The notion that group interventions have a greater impact than individual approaches is reflected in the results of one included study that found a group intervention produced a large effect size, compared to the moderate effect produced by same intervention, but implemented in a one-on-one setting [44]. This highlights the need for further investigation as to the ideal setting for pragmatic language interventions and the factors that mediate change. Individual interventions could potentially be enhanced through the inclusion of techniques used in the group interventions, but a knowledge gap is evident in the included studies as to the factors that may have mediated the changes measured in each intervention. Data from much larger participant samples than those included in this review would need to be collected in order to reliably analyse mediating and moderating factors. However, if the mediating and moderating factors that positively influence intervention outcomes were known then those factors that had largest influence on change could be incorporated into individual interventions in order to enhance their effectiveness.

Notably, groups were comprised exclusively of peers with ASD in all interventions, with the exception of SENSE Theater which included typically developing peers [45]. This is contrasted by a systematic review of peer-mediated interventions for children with ASD, in which a majority of studies (34 of the 42) included peers without a disability [64]. There is emerging literature suggesting that the use of typically developing peers in group interventions increases the social interactions of children and adolescents with ASD, and aid in skill maintenance and generalisation in the long term [65]. It is possible then, that the inclusion of typically developing peers has the potential to further increase the effectiveness of the group interventions included in this review; clearly this is an avenue worth exploring.

Skill generalisation is a continuing problem for social interaction interventions for children with ASD [66]. Decontextualised learning has been identified as a barrier to generalisation in other social skill interventions for children with ASD and recommendations such as home-based practice, parent involvement in therapy, and practice with a variety of people and settings have been made to aid generalisation [67–69]. A majority of included pragmatic language interventions (71%) included in this review were set in the clinic and approximately half of the interventions (11) included strategies for generalisation, such as the involvement of parents in interventions and the inclusion of out-of-session practice. The clinic was found to be the most effective setting when compared to home or school, and even though strategies to enhance skill generalisation were included in most of the clinic-based interventions, little is known about whether these strategies were effective. Outcome measurement often assessed pragmatic language in the context in which the intervention was administered or via a decontextualised assessment instrument, so conclusions cannot be drawn as to the generalisability of skills following these interventions. This highlights the need for researchers to consider including assessments in their investigations that capture behavioural observations of pragmatic language skills in varying contexts. Additionally, clinic-based interventions can be inaccessible to some families because of financial or logistical limitations, and there can be a limited availability of therapists in some locations, particularly in rural settings. These factors highlight the need for further development and research to enhance the effectiveness of school based interventions, or programs that increase the effectiveness of parents as interventionists in the home.

This review found that the person(s) of treatment focus was the only variable identified as being a significant mediating factor in the meta-regression. Interventions that focused on treating the child as well as coaching parents in intervention techniques produced the greatest effect, with some of these interventions occurring in the home, and others occurring in the clinic. These results are mirrored in a recent review of spoken language interventions for children with ASD. The review found approaches that included both the clinician and parent in the delivery of therapy produced a significant, moderate effect in comparison to approaches delivered by the clinician or parent only [15]. Results from both reviews are in contrast to the findings of a review of parent-mediated interventions for children with ASD. Specifically, the review of parent-mediated interventions found mixed results as to the effectiveness of such approaches in improving language and social communication in young children with ASD [70]. However, the importance of including parents in interventions for children with ASD is also recognised in the same review due to a caregiver’s capacity to provide intervention early, and across a variety of environments and people.

Interestingly, one intervention included in this systematic review, investigated the effectiveness of parent training seminars without the child being present [56]. That study produced a negligible effect in comparison to other interventions that were delivered directly to the child or child-parent dyad (see Fig 4). If parents are to implement interventions in the home to enhance treatment efficacy, then generic training seminars may not be the ideal approach. Clinicians should also observe the parent-child interaction in order to customise training to the family, and provide parents with specific feedback on progress. The rationale provided by the authors for studying a caregiver-training only intervention was to provide assistance to *low resourced* families who might not otherwise be able to access intervention services. Given the negligible effect of this delivery model, further investigation of caregiver-training approaches is needed. Establishing the appropriate balance between the clinician and parent components of interventions could increase effectiveness and accessibility to services. Clearly, there is a need for further research in the area of parent-mediated interventions for improving pragmatic language in order for stronger conclusions to be drawn.

Pragmatic language encompasses a complex skill set; the execution of which needs to be constantly adjusted in dynamic social environments. As such, assessing pragmatic language is challenging for clinicians and researchers alike. In assessing pragmatic language outcomes, 10 studies included in this review utilised parent report rating scales or lab-based assessments administered to the child. The results of the meta-analysis indicate that a larger effect size is likely to be detected when pragmatic language is measured through these types of measures when compared to observational measures. The potential introduction of bias through the use of parent questionnaires has already been discussed in this paper due to the inability to blind caregivers to treatment conditions. Additionally, the structured nature of standardised lab-based assessments fails to capture the complex dynamics of the social context and is often not the ideal assessment medium for children with ASD. Eleven included studies utilised observational ratings of pragmatic language skills. While these produced only a small effect size in comparison to other types of outcome measures, the ecological validity of these outcomes measures is recognised and perhaps provide a truer indication of the effect of the interventions studied. However, if researchers and clinicians are to use observational measures of pragmatic language, further investigation of the psychometric properties of available instruments is required. While the inter-rater reliability of observational measures is commonly reported in the included studies, other psychometric properties such as, internal consistency, validity and responsiveness, of the measures is mostly unknown.

A majority of the interventions reviewed (14 out of 20) targeted non-verbal communication, a hallmark impairment of ASD [71]. Skills were usually targeted in isolation with just seven interventions targeting a combination of pragmatic language skills. With the expanding definition of pragmatic language comes a need for interventions to target a wider skill set, especially in the over 5-year age group. No one intervention included in this review targeted all of pragmatic language skills, and additionally, none of the studies targeted the skills of executive function or negotiation. Targeting skills in isolation neglects the dynamic and complex nature of social interactions. It is possible that interventions that target one skill show a large effect, but are not as clinically beneficial as more holistic approaches that obtain smaller effects. More research is required into the effectiveness of interventions that target a more comprehensive skill set for pragmatic language.

Only one study differentiated groups by the presence or absence of an intellectual disability [49]. The intervention group with participants who did not have an intellectual disability demonstrated a large treatment effect. This is contrasted against the moderate effect measured in the intervention group of children with ASD with an intellectual disability who received the same intervention. This could mean that children without an intellectual disability gain more from pragmatic language interventions; however, due caution needs to be exercised here and more research is required comparing the cognitive profiles of children with ASD and the impact this has on intervention effectiveness. These findings also emphasise the heterogeneity in autism profiles and the need to consider factors that might mediate an intervention’s effect in order to make interventions as beneficial as possible.

The longitudinal benefits of the included interventions are mostly unknown. Follow-up data were reported in nine papers with times ranging from 5-weeks to 12-months post-intervention. Given that individuals with ASD experience pragmatic language impairments into adulthood [14], there is a need for researchers to track the benefits of interventions overextended time frames to evaluate their effectiveness in improving long-term social functioning.

Finally, results of the meta-analysis showed that treatment effects were greatest when comparison groups received no treatment (i.e., waitlisted controls), and the effect of tailored pragmatic language interventions was negligible in comparison to the treatment as usual control conditions. Again, these results are mirrored the findings of a review of spoken language interventions for children with ASD; targeted interventions were no more effective in improving spoken language than comprehensive ASD interventions [15]. Intervention approaches for improving pragmatic language, trialled with children with ASD show some promise; however, factors that might mediate greater change and the generalisation of skills need further investigation. In summary, we need a greater understanding of: a) how cognitive and language profiles influence treatment effects; b) the most effective intervention setting and intervention agents to achieve large effects; and c) the inclusion of more strategies to enhance skill generalisation.

## Limitations

Great care was taken during the process of this review in order to minimise the introduction of bias. A comprehensive search was conducted including relevant databases alongside a number of professional and academic information sources. Abstract screening for study selection and ratings of methodological quality were conducted by two independent researchers with acceptable levels of interrater reliability. Despite its methodological rigour, this review is subject to a number of limitations. Quasi-experimental design studies and single case experimental designs were excluded from the review. The choice to include randomised study designs only when evaluating interventions for children with ASD could confound results given the potential for high levels of heterogeneity in participant samples. The included studies are also at risk of bias due to limitations in methodological design or reporting. The potential for within-group heterogeneity in samples of children with ASD, coupled with incomplete control for confounding variables and inadequate blinding, somewhat limits the conclusions that can be generalised to the broader population of children with ASD. With the exception of participant age, this study was also unable to address whether other participant characteristics (e.g., expressive or receptive language ability, autism symptom severity, cognitive ability) impacted on the effect of the included interventions. This was due to inadequate reporting of participant demographic and diagnostic variables.

## Conclusions

The consequences of the social communication impairments in children with ASD are far reaching and life-long, and tailored pragmatic language interventions have the potential to reduce these impacts for children with ASD. This review of pragmatic language interventions for children with ASD found a number of promising approaches. Findings of this meta-analysis suggest that the person(s) of focus is a significant mediator of intervention effect, but the age of participants is not, suggesting that regardless of age, the child with ASD and their parent must be actively included in an intervention in order to maximise benefits. Further, group interventions appear to be more effective than those delivered one-on-one, and the inclusion of typically developing peers may have the potential to increase the effectiveness of group interventions. At this point, the generalisation of pragmatic language skills outside of the clinical context and longitudinal effects of pragmatic language interventions for children with ASD are largely unknown. There is a need for more studies that investigate: the most effective dosage of these intervention approaches; intervention effectiveness when confounding variables such as language competence or intellectual ability are controlled for; and the development of interventions targeting pragmatic language skills in adolescents with ASD. The bias introduced into a number of studies via the use of parent rated measures of pragmatic language highlights the need for further development in the area of pragmatic language measurement. Instruments that capture the complex nature of the social interactions are required so that researchers and clinicians can obtain unbiased measurements of pragmatic language competence to assess change following intervention as well as skill generalisation.

## Supporting information

S1 Table PRISMA checklist. (DOCX)

S2 Table Search terms. (DOCX)

## References

[1] Paul R, Norbury C. Language disorders from infancy through adolescence: Listening, speaking, reading, writing, and communicating. St. Louis, MO: Elsevier Health Sciences; 2012.

[2] Prutting C, Kirchner. A clinical apraisal of the pragmatic aspects of language. J Speech Hear Disord. 1987;52:105–19. 3573742 10.1044/jshd.5202.105

[3] Adams C, Baxendale B, Lloyd J, Aldred C. Pragmatic language impairment: Case studies of social and pragmatic language therapy. Child Lang Teach Ther. 2005;21(3):227–50.

[4] Snow CE, Pan BA, Imbens-Bailey A, Herman J. Learning how to say what one means: A longitudinal study of children's speech act use. Social Development. 1996;5(1):56–84.

[5] Mundy P, Sigman M, Kasari C. A longitudinal study of joint attention and langauge development in autistic children. J Autism Dev Disord. 1990;20(1):115–28. 2324051 10.1007/BF02206861

[6] Tager-Flusberg H, Kasari C. Minimally verbal school-aged children with autism spectrum disorder: the neglected end of the spectrum. Autism Res. 2013;6(6):468–78. 10.1002/aur.1329 24124067 PMC3869868

[7] Bono MA, Daley T, Sigman M. Relations among joint attention, amount of intervention and language gain in autism. J Autism Dev Disord. 2004;34(5):495–505. 15628604 10.1007/s10803-004-2545-x

[8] Cordier R, Munro N, Wilkes-Gillan S, Speyer R, Pearce WM. Reliability and validity of the Pragmatics Observational Measure (POM): A new observational measure of pragmatic language for children. Res Dev Disabil. 2014;35(7):1588–98. 10.1016/j.ridd.2014.03.050 24769431

[9] Aldred C, Green J, Adams C. A new social communication intervention for children with autism: Pilot randomised controlled treatment study suggesting effectiveness. Journal of Child Psychology and Psychiatry. 2004;45(8):1420–30. 10.1111/j.1469-7610.2004.00848.x 15482502

[10] Ciccia AH, Turkstra LS. Cohesion, communication burden, and response adequacy in adolescent conversations. Advances in Speech Language Pathology. 2002;4(1):1–8.

[11] Paul R, Orlovski SM, Marcinko HC, Volkmar F. Conversational behaviors in youth with high-functioning ASD and Asperger syndrome. J Autism Dev Disord. 2009;39(1):115–25. 10.1007/s10803-008-0607-1 18607708 PMC2819316

[12] Bauminger N, Kasari C. Loneliness and friendship in high-functioning children with autism. Child Dev. 2000:447–56. 10834476 10.1111/1467-8624.00156

[13] Locke J, Ishijima EH, Kasari C, London N. Loneliness, friendship quality and the social networks of adolescents with high-functioning autism in an inclusive school setting. Journal of Research in Special Educational Needs. 2010;10(2):74–81.

[14] Whitehouse AJ, Watt HJ, Line EA, Bishop DV. Adult psychosocial outcomes of children with specific language impairment, pragmatic language impairment and autism. Int J Lang Commun Disord. 2009;44(4):511–28. 10.1080/13682820802708098 19340628 PMC2835860

[15] Hampton L, Kaiser A. Intervention effects on spoken-language outcomes for children with autism: a systematic review and meta-analysis. J Intellect Disabil Res. 2016;60(5):444–63. 10.1111/jir.12283 27120988

[16] Kmet LM, Lee RC, Cook LS. Standard quality assessment criteria for evaluating primary research papers from a variety of fields. Alberta, Canada: Alberta Heritage Foundation for Medical Research; 2004.

[17] Lee L, Packer TL, Tang SH, Girdler S. Self-management education programs for age-related macular degeneration: A systematic review. Australas J Ageing. 2008;27(4):170–6. 10.1111/j.1741-6612.2008.00298.x 19032617

[18] Millard T, Elliott J, Girdler S. Self-management education programs for people living with HIV/AIDS: a systematic review. AIDS Patient Care STDS. 2013;27(2):103–13. 10.1089/apc.2012.0294 23298279

[19] Hoffmann TC, Glasziou PP, Boutron I, Milne R, Perera R, Moher D, et al Better reporting of interventions: template for intervention description and replication (TIDieR) checklist and guide. Br Med J. 2014;348:g1687. 24609605 10.1136/bmj.g1687

[20] Hedges LV, Pigott TD. The power of statistical tests for moderators in meta-analysis. Psychol Methods. 2004;9(4):426 10.1037/1082-989X.9.4.426 15598097

[21] Higgins JP, Thompson SG, Deeks JJ, Altman DG. Measuring inconsistency in meta-analyses. BMJ. 2003;327(7414):557–60. 10.1136/bmj.327.7414.557 12958120 PMC192859

[22] Cohen J. Statistical power analysis for the behavioral sciences. 2nd ed Hillsdale, NJ: Earlbaum; 1988.

[23] Gattino GS, dos Santos Riesgo R, Longo D, Loguercio Leite JC, Faccini LS. Effects of relational music therapy on communication of children with autism: A randomized controlled study. Nordic Journal of Music Therapy. 2011;20(2):142–54.

[24] Ichikawa K, Takahashi Y, Ando M, Anme T, Ishizaki T, Yamaguchi H, et al TEACCH-based group social skills training for children with high-functioning autism: A pilot randomized controlled trial. Biopsychosoc Med. 2013;7(1):14 10.1186/1751-0759-7-14 24083413 PMC3850504

[25] Kasari C, Rotheram-Fuller E, Locke J, Gulsrud A. Making the connection: Randomized controlled trial of social skills at school for children with autism spectrum disorders. Journal of Child Psychology and Psychiatry. 2012;53(4):431–9. 10.1111/j.1469-7610.2011.02493.x 22118062 PMC3238795

[26] Lerner MD, Mikami AY. A preliminary randomized controlled trial of two social skills interventions for youth with high-functioning autism spectrum disorders. Focus Autism Other Dev Disabl. 2012;27(3):147–57.

[27] Wong VC, Kwan QK. Randomized controlled trial for early intervention for autism: A pilot study of the Autism 1-2-3 Project. J Autism Dev Disord. 2010;40(6):677–88. 10.1007/s10803-009-0916-z 20020319

[28] Houghton K, Schuchard J, Lewis C, Thompson CK. Promoting child-initiated social-communication in children with autism: Son-Rise Program intervention effects. J Commun Disord. 2013;46(5–6):495–506. Language: English. Entry Date: 20140110. Revision Date: 20140620. Publication Type: journal article. 10.1016/j.jcomdis.2013.09.004 24209427

[29] McFadden B, Kamps D, Heitzman-Powell L. Social communication effects of peer-mediated recess intervention for children with autism. Res Autism Spectr Disord. 2014;8(12):1699–712. 10.1016/j.rasd.2014.08.015 26312064 PMC4547561

[30] McMahon CM, Vismara LA, Solomon M. Measuring changes in social behavior during a social skills intervention for higher-functioning children and adolescents with autism spectrum disorder. J Autism Dev Disord. 2013;43(8):1843–56. 10.1007/s10803-012-1733-3 23239098 PMC3676723

[31] Oosterling I, Visser J, Swinkels S, Rommelse N, Donders R, Woudenberg T, et al Randomized controlled trial of the focus parent training for toddlers with autism: 1-year outcome. J Autism Dev Disord. 2010;40(12):1447–58. 10.1007/s10803-010-1004-0 20440639 PMC2980624

[32] Radley KC, Ford WB, Battaglia AA, McHugh MB. The effects of a social skills training package on social engagement of children with autism spectrum disorders in a generalized recess setting. Focus Autism Other Dev Disabl. 2014;29(4):216–29.

[33] Shire SY, Goods K, Shih W, Distefano C, Kaiser A, Wright C, et al Parents' adoption of social communication intervention strategies: Families including children with autism spectrum disorder who are minimally verbal. J Autism Dev Disord. 2014;45(6):1712–24. 10.1007/s10803-014-2329-x PMC4442706 25475363

[34] Wetherby AM, Guthrie W, Woods J, Schatschneider C, Holland RD, Morgan L, et al Parent-implemented social intervention for toddlers with autism: An RCT. Pediatrics. 2014;134(6):1084–93. Language: English. Entry Date: 20141219. Revision Date: 20141226. Publication Type: journal article. 10.1542/peds.2014-0757 25367544 PMC4243066

[35] Adams C, Lockton E, Freed J, Gaile J, Earl G, McBean K, et al The Social Communication Intervention Project: A randomized controlled trial of the effectiveness of speech and language therapy for school-age children who have pragmatic and social communication problems with or without autism spectrum disorder. Int J Lang Commun Disord. 2012;47(3):233–44. 10.1111/j.1460-6984.2011.00146.x 22512510

[36] Kamps D, Thiemann-Bourque K, Heitzman-Powell L, Schwartz I, Rosenberg N, Mason R, et al A comprehensive peer network intervention to improve social communication of children with autism spectrum disorders: A randomized trial in kindergarten and first grade. J Autism Dev Disord. 2014;45(6):1809–24. 10.1007/s10803-014-2340-2 PMC4442739 25510450

[37] Donaldson AL. Siblings of children with ASD: Promoting social communication. Perspectives on Language Learning and Education. 2015;22(1):31–8.

[38] Lopata C, Thomeer ML, Volker MA, Toomey JA, Nida RE, Lee GK, et al RCT of a manualized social treatment for high-functioning autism spectrum disorders. J Autism Dev Disord. 2010;40(11):1297–310. 10.1007/s10803-010-0989-8 20232240

[39] Lopata C, Thomeer ML, Rodgers JD, Donnelly JP, McDonald CA. RCT of mind reading as a component of a psychosocial treatment for high-functioning children with ASD. Res Autism Spectr Disord. 2016;21:25–36.

[40] Thomeer ML, Smith RA, Lopata C, Volker MA, Lipinski AM, Rodgers JD, et al Randomized controlled trial of Mind Reading and in vivo rehearsal for high-functioning children with ASD. J Autism Dev Disord. 2015;45(7):2115–27. 10.1007/s10803-015-2374-0 25643864

[41] Beaumont R, Sofronoff K. A multi-component social skills intervention for children with Asperger syndrome: The Junior Detective Training Program. Journal of Child Psychology and Psychiatry. 2008;49(7):743–53. 10.1111/j.1469-7610.2008.01920.x 18503531

[42] Casenhiser DM, Shanker SG, Stieben J. Learning through interaction in children with autism: Preliminary data from asocial-communication-based intervention. Autism. 2013;17(2):220–41. 10.1177/1362361311422052 21949005

[43] Casenhiser DM, Binns A, McGill F, Morderer O, Shanker SG. Measuring and supporting language function for children with autism: Evidence from a randomized control trial of a social-interaction-based therapy. J Autism Dev Disord. 2015;45(3):846–57. 10.1007/s10803-014-2242-3 25234481

[44] Roberts J, Williams K, Carter M, Evans D, Parmenter T, Silove N, et al A randomised controlled trial of two early intervention programs for young children with autism: Centre-based with parent program and home-based. Res Autism Spectr Disord. 2011;5(4):1553–66.

[45] Corbett BA, Key AP, Qualls L, Fecteau S, Newsom C, Coke C, et al Improvement in social competence using a randomized trial of a theatre intervention for children with autism spectrum disorder. J Autism Dev Disord. 2015:1–15. 26419766 10.1007/s10803-015-2600-9 PMC5633031

[46] DeRosier ME, Swick DC, Davis NO, McMillen JS, Matthews R. The efficacy of a social skills group intervention for improving social behaviors in children with high functioning autism spectrum disorders. J Autism Dev Disord. 2011;41(8):1033–43. 10.1007/s10803-010-1128-2 21042870

[47] Fletcher-Watson S, Petrou A, Scott-Barrett J, Dicks P, Graham C, O’Hare A, et al A trial of an iPad intervention targeting social communication skills in children with autism. Autism. 2015. 10.1177/1362361315605624 PMC5015758 26503990

[48] Gabriels RL, Pan Z, Dechant B, Agnew JA, Brim N, Mesibov G. Randomized controlled trial of therapeutic horseback riding in children and adolescents with autism spectrum disorder. J Am Acad Child Adolesc Psychiatry. 2015;54(7):541–9. 10.1016/j.jaac.2015.04.007 26088658 PMC4475278

[49] Hopkins IM, Gower MW, Perez TA, Smith DS, Amthor FR, Wimsatt F, et al Avatar assistant: Improving social skills in students with an ASD through a computer-based intervention. J Autism Dev Disord. 2011;41(11):1543–55. 10.1007/s10803-011-1179-z 21287255

[50] Hauck M, Fein D, Waterhouse L, Feinstein C. Social initiations by autistic children to adults and other children. J Autism Dev Disord. 1995;25(6):579–95. 8720028 10.1007/BF02178189

[51] Kaale A, Smith L, Sponheim E. A randomized controlled trial of preschool-based joint attention intervention for children with autism. J Child Psychol Psychiatry. 2012;53(1):97–105. 10.1111/j.1469-7610.2011.02450.x 21883204

[52] Kaale A, Fagerland MW, Martinsen EW, Smith L. Preschool-based social communication treatment for children with autism: 12-month follow-up of a randomized trial. J Am Acad Child Adolesc Psychiatry. 2014;53(2):188–98. 10.1016/j.jaac.2013.09.019 24472253

[53] Kasari C, Freeman S, Paparella T. Joint attention and symbolic play in young children with autism: A randomized controlled intervention study. Journal of Child Psychology and Psychiatry. 2006;47(6):611–20. 10.1111/j.1469-7610.2005.01567.x 16712638

[54] Lawton K, Kasari C. Brief report: Longitudinal improvements in the quality of joint attention in preschool children with autism. J Autism Dev Disord. 2012;42(2):307–12. 10.1007/s10803-011-1231-z 22187107 PMC4194068

[55] Kasari C, Gulsrud AC, Wong C, Kwon S, Locke J. Randomized controlled caregiver mediated joint engagement intervention for toddlers with autism. J Autism Dev Disord. 2010;40(9):1045–56. 10.1007/s10803-010-0955-5 20145986 PMC2922697

[56] Kasari C, Lawton K, Shih W, Barker TV, Landa R, Lord C, et al Caregiver-mediated intervention for low-resourced preschoolers with autism: An RCT. Pediatrics. 2014;134(1):e72–e9. 10.1542/peds.2013-3229 24958585 PMC4531276

[57] Kim J, Wigram T, Gold C. The effects of improvisational music therapy on joint attention behaviors in autistic children: A randomized controlled study. J Autism Dev Disord. 2008;38(9):1758–66. 10.1007/s10803-008-0566-6 18592368

[58] Ryan C, Charragain CN. Teaching emotion recognition skills to children with autism. J Autism Dev Disord. 2010;40(12):1505–11. 10.1007/s10803-010-1009-8 20386975

[59] Soorya LV, Siper PM, Beck T, Soffes S, Halpern D, Gorenstein M, et al Randomized comparative trial of a social cognitive skills group for children with autism spectrum disorder. J Am Acad Child Adolesc Psychiatry. 2015;54(3):208–16. 10.1016/j.jaac.2014.12.005 25721186 PMC4346205

[60] Baron-Cohen S. Mind reading: The interactive guide to emotions. London UK: Jessica Kingsley Publishers; 2003.

[61] Gluud LL. Bias in clinical intervention research. Am J Epidemiol. 2006;163(6):493–501. 10.1093/aje/kwj069 16443796

[62] Liberati A, Altman D, Tetzlaff J, Mulrow C, Gøtzsche P, Ioannidis J, et al The PRISMA statement for reporting systematic and meta-analyses of studies that evaluate interventions: explanation and elaboration. PLoS Med. 1999;6(7):1–28. 10.1371/journal.pmed.1000100 PMC2707010 19621070

[63] Gerenberg M. Promoting resilience in children and youth: Preventive interventions and their interface with neurescıence. New York Academy of Sciences. 2006;1094:139–50. 10.1196/annals.1376.013 17347347

[64] Chan JM, Lang R, Rispoli M, O’Reilly M, Sigafoos J, Cole H. Use of peer-mediated interventions in the treatment of autism spectrum disorders: A systematic review. Res Autism Spectr Disord. 2009;3(4):876–89.

[65] Watkins L, O’Reilly M, Kuhn M, Gevarter C, Lancioni GE, Sigafoos J, et al A review of peer-mediated social interaction interventions for students with autism in inclusive settings. J Autism Dev Disord. 2015;45(4):1070–83. 10.1007/s10803-014-2264-x 25272953

[66] Rao PA, Beidel DC, Murray MJ. Social skills interventions for children with Asperger’s syndrome or high-functioning autism: A review and recommendations. J Autism Dev Disord. 2008;38(2):353–61. 10.1007/s10803-007-0402-4 17641962

[67] Kransy L, Williams BJ, Provencal S, Ozonoff S. Social skills interventions for the autism spectrum: Essential ingredients and a model curriculum. Child and Adolescent Psychiatric Clinics. 2002;12:107–22. 10.1016/s1056-4993(02)00051-2 12512401

[68] Spence S. Social skills training with children and young people: Theory, evidence and practice. Child and Adolescent Mental Health. 2003;8(2):84–96. 10.1111/1475-3588.00051 32797550

[69] Williams White S, Keonig K, Scahill L. Social skills development in children with autism spectrum disorders: A review of the intervention research. J Autism Dev Disord. 2007;37(10):1858–68. 10.1007/s10803-006-0320-x 17195104

[70] Oono IP, Honey EJ, McConachie H. Parent-mediated early intervention for young children with autism spectrum disorders (ASD). Evidence-Based Child Health: A Cochrane Review Journal. 2013;8(6):2380–479. 10.1002/14651858.CD009774.pub2 PMC11831248 23633377

[71] Chiang C-H, Soong W-T, Lin T-L, Rogers SJ. Nonverbal communication skills in young children with autism. J Autism Dev Disord. 2008;38(10):1898–906. 10.1007/s10803-008-0586-2 18491223 PMC4951089

