# Supplementary material for: A systematic review of pragmatic language interventions for children with autism spectrum disorder
Source: PLoS One. 2017 Apr 20;12(4):e0172242. doi: 10.1371/journal.pone.0172242 (PMC5398499; doi:10.1371/journal.pone.0172242)
Supplement: S2 Table — (DOCX) [file pone.0172242.s002.docx]

**S2 Table. Search Terms**

|  | **Database and Search Terms** | **Limitations** |
| --- | --- | --- |
| **Subject Headings** | **CINAHL**: ((MH "Speech and Language Assessment/CL/ED/EV/MT/PF/TD/UT") OR (MH "Rehabilitation, Speech and Language/CL/UT/TD/PF/MT/EV/ED") OR (MH "Nonverbal Communication/CL/ED/EV/MT/PC/TD/UT") OR (MH "Impaired Verbal Communication (NANDA)/EV/UT") OR (MH "Communicative Disorders/CL/DI/ED/TH/RH/PF/PC/TD") OR (MH "Social Behavior/CL/ED/EV/PC/TD") OR (MH "Social Behavior Disorders/CL/DI/ED/PC/PF/TH/TD/RH") OR (MH "Social Skills/CL/ED/EV/PC/TD") OR (MH "Communication/CL/ED/EV/MT/PC/TD/UT") OR (MH "Communication Methods, Total/CL/ED/EV/MT/PF/TD/UT") OR (MH "Impaired Verbal Communication (NANDA)/EV/UT") OR (MH "Communication Skills/ED/CL/EV/MT/PC/TD/UT") OR (MH "Communication Impairment (Saba CCC)/ED/EV/TH/UT") OR (MH "Communication Ability (Iowa NOC)/EV/UT") OR (MH "Communication: Receptive Ability (Iowa NOC)/EV/UT") OR (MH "Communication: Expressive Ability (Iowa NOC)/EV/UT") OR (MH "Communicative Disorders/CL/ED/PF/PC/TD/TH/RH") OR (MH "Language Development/ED/EV/PC/TD") OR (MH "Speech and Language Assessment/CL/ED/EV/MT/TD/UT") OR (MH "Research, Speech-Language-Hearing Therapy/CL/ED/EV/MT/TU/TD/UT") OR (MH "Verbal Behavior/ED/EV/PC/TD") OR (MH "Language/CL/ED/EV/TD/UT/MT/PC") OR (MH "Language Tests/ED/EV/MT/PF/TU/TD/UT/CL") OR (MH "Language Therapy/CL/ED/EV/MT/PF/UT/TD") OR (MH "Language Disorders/CL/ED/TD/TH/RH/PF/PC") OR (MH "Speech-Language Pathology/CL/ED/EV/MT/PF/TD/UT") OR (MH "Speech and Language (Omaha)/EV/UT") OR (MH "Speech Therapy/CL/ED/EV/MT/PF/TD/UT")) AND ((MH "Autistic Disorder") OR (MH "Child Development Disorders, Pervasive") OR (MH "Pervasive Developmental Disorder-Not Otherwise Specified") OR (MH "Asperger Syndrome") OR (MH "Rett Syndrome")) AND (pragmatic* OR paralinguistic* OR (TI(social AND communication) OR AB(social AND communication)) | Narrow by Subject Age: all infant; adolescent:13-18 years; child preschool: 2-5 years; child: 6-12 years; all child |
|  | **Embase:** (social learning/ OR social competence/ OR social behavior/ OR nonverbal communication/ OR social adaptation/ OR communication skill/ OR language ability/ OR nonverbal communication/ OR verbal communication/ OR communication disorder/di, dm, pc, rh, th [Diagnosis, Disease Management, Prevention, Rehabilitation, Therapy] OR language ability/ OR language delay/ OR language development/ OR language disability/ OR language processing/ OR verbal behavior/ OR verbal communication/ OR language/ OR language test/ OR OR speech rehabilitation/ OR speech therapy/ OR developmental language disorder/di, pc, rh, th [Diagnosis, Prevention, Rehabilitation, Therapy]) AND (autism/ OR "pervasive developmental disorder not otherwise specified"/ OR Rett syndrome/ OR childhood disintegrative disorder/) AND (pragmatic* OR paralinguistic* OR (social AND communication)).mp. [mp=title, abstract, subject headings, heading word, drug trade name, original title, device manufacturer, drug manufacturer, device trade name, keyword] | English; infant <to one year> or preschool child <1 to 6 years> or school child <7 to 12 years> or adolescent <13 to 17 years> |
|  | **Eric: (**DE "Autism" OR DE "Pervasive Developmental Disorders" OR DE "Asperger Syndrome") AND (DE “Pragmatics” OR DE “Paralinguistics”) | English |
|  | **PsycINFO**: (autism/ OR aspergers syndrome/ OR pervasive developmental disorders/ OR rett syndrome/) AND (pragmatics/) | English; infant <to one year> or preschool child <1 to 6 years> or school child <7 to 12 years> or adolescent <13 to 17 years> |
|  | **PubMed:** (Therapy/education"[Mesh] OR "Speech Therapy/instrumentation"[Mesh] OR "Speech Therapy/methods"[Mesh] OR "Speech Therapy/psychology"[Mesh] OR "Speech Therapy/trends"[Mesh] OR "Speech Therapy/utilization"[Mesh] OR "Specific Language Impairment 4" [Supplementary Concept] OR "Child Language"[Mesh] OR "Language Development/classification"[Mesh] OR "Language Development/prevention and control"[Mesh] OR "Language Development/psychology"[Mesh] OR "Language Development/therapy"[Mesh] OR "Language Development Disorders/classification"[Mesh] OR "Language Development Disorders/diagnosis"[Mesh] OR "Language Development Disorders/prevention and control"[Mesh] OR "Language Development Disorders/psychology"[Mesh] OR "Language Development Disorders/rehabilitation"[Mesh] OR "Language Development Disorders/therapy"[Mesh] OR "Language Disorders/classification"[Mesh] OR "Language Disorders/diagnosis"[Mesh] OR "Language Disorders/education"[Mesh] OR "Language Disorders/prevention and control"[Mesh] OR "Language Disorders/psychology"[Mesh] OR "Language Disorders/rehabilitation"[Mesh] OR "Language Disorders/therapy"[Mesh] OR "Language Tests/classification"[Mesh] OR "Language Tests/instrumentation"[Mesh] OR "Language Tests/methods"[Mesh] OR "Language Therapy/classification"[Mesh] OR "Language Therapy/education"[Mesh] OR "Language Therapy/instrumentation"[Mesh] OR "Language Therapy/methods"[Mesh] OR "Language Therapy/psychology"[Mesh] OR "Language Therapy/therapeutic use"[Mesh] OR "Language Therapy/trends"[Mesh] OR "Rehabilitation of Speech and Language Disorders/classification"[Mesh] OR "Rehabilitation of Speech and Language Disorders/education"[Mesh] OR "Rehabilitation of Speech and Language Disorders/instrumentation"[Mesh] OR "Rehabilitation of Speech and Language Disorders/methods"[Mesh] OR "Rehabilitation of Speech and Language Disorders/psychology"[Mesh] OR "Rehabilitation of Speech and Language Disorders/therapeutic use"[Mesh] OR "Rehabilitation of Speech and Language Disorders/therapy"[Mesh] OR "Rehabilitation of Speech and Language Disorders/trends"[Mesh] OR "Rehabilitation of Speech and Language Disorders/utilization"[Mesh] OR "Speech-Language Pathology/classification"[Mesh] OR "Speech-Language Pathology/education"[Mesh] OR "Speech-Language Pathology/instrumentation"[Mesh] OR "Speech-Language Pathology/methods"[Mesh] OR "Speech-Language Pathology/trends"[Mesh] OR "Language/classification"[Mesh] OR "Language/diagnosis"[Mesh] OR "Language/education"[Mesh] OR "Language/instrumentation"[Mesh] OR "Language/methods"[Mesh] OR "Language/psychology"[Mesh] OR "Language/therapy"[Mesh] OR "Language/trends"[Mesh] OR "Verbal Behavior/classification"[Mesh] OR "Verbal Behavior/education"[Mesh] OR "Verbal Behavior/psychology"[Mesh] OR "Verbal Behavior/therapy"[Mesh] OR "Communication Barriers"[Mesh] OR "Communication Methods, Total"[Mesh] OR "Communication Disorders/classification"[Mesh] OR "Communication Disorders/diagnosis"[Mesh] OR "Communication Disorders/education"[Mesh] OR "Communication Disorders/prevention and control"[Mesh] OR "Communication Disorders/psychology"[Mesh] OR "Communication Disorders/rehabilitation"[Mesh] OR "Communication Disorders/therapy"[Mesh] OR "Manual Communication"[Mesh] OR "Communication/classification"[Mesh] OR "Communication/diagnosis"[Mesh] OR "Communication/education"[Mesh] OR "Communication/instrumentation"[Mesh] OR "Communication/methods"[Mesh] OR "Communication/prevention and control"[Mesh] OR "Communication/psychology"[Mesh] OR "Communication/therapy"[Mesh] OR "Communication/trends"[Mesh] OR "Social Skills"[Mesh] OR "Social Behavior"[Mesh] OR "Social Behavior Disorders"[Mesh] OR "Speech Production Measurement/instrumentation"[Mesh] OR "Speech Production Measurement/methods"[Mesh] OR "Speech Production Measurement/psychology"[Mesh] OR "Speech Production Measurement/therapy"[Mesh] OR "Nonverbal Communication/classification"[Mesh] OR "Nonverbal Communication/diagnosis"[Mesh] OR "Nonverbal Communication/education"[Mesh] OR "Nonverbal Communication/instrumentation"[Mesh] OR "Nonverbal Communication/methods"[Mesh] OR "Nonverbal Communication/prevention and control"[Mesh] OR "Nonverbal Communication/psychology"[Mesh] OR "Nonverbal Communication/therapy"[Mesh] OR "Nonverbal Communication/trends"[Mesh] OR "Language Development Disorders"[Mesh] OR "Pragmatic Clinical Trial" [Publication Type] OR "Pragmatic Clinical Trials as Topic"[Mesh]) AND ("Autistic Disorder"[Mesh] OR "Child Development Disorders, Pervasive"[Mesh] OR "Rett Syndrome"[Mesh] OR "Asperger Syndrome"[Mesh]) AND ((pragmatic* OR paralinguistic*) OR (social AND communication Field: Title/Abstract)) AND (English[lang] AND (infant[MeSH] OR child[MeSH] OR adolescent[MeSH])) | English; Child: birth-18 years |
| **Free Text Words** | **CINAHL:** (child* OR toddler* OR infant* OR schoolchild* OR youth* OR baby OR babies OR pediatr* OR paediatr* OR neonat* OR newborn* OR postneonat* OR postnat* OR suckling* OR juvenile* OR adolescent* OR teenager* OR teen-ager* OR pubescent* OR pubertal OR youngster* OR minor*) AND (autism OR autistic OR ASD OR PDD OR PDD-NOS OR pervasive OR Asperger OR Rett OR (childhood AND disintegrative AND disorder*)) AND ((social AND communication) OR (pragmatic* OR paralinguistic*)) | *Initial search:* Publication date from 2014/04/08 to 2016/05/31; Field: Title/Abstract) |
|  | **Embase*:*** *As per CINAHL Free Text* | 2015 to current |
|  | **Eric:** *As per CINAHL Free Text* | *Initial search:* Publication date from 2014/04/08 to 2016/05/31; Field: Title/Abstract) |
|  | **PsycINFO:** *As per CINAHL Free Text* | Publication year 2015-2016 |
|  | **PubMed:** *As per CINAHL Free Text* | Publication date from 2014/04/08 to 2016/05/14; Field: Title/Abstract |
